# Bayesian and Discriminative Models for Active Visual Perception across Saccades

**DOI:** 10.1523/ENEURO.0403-22.2023

**Published:** 2023-07-20

**Authors:** Divya Subramanian, John M. Pearson, Marc A. Sommer

**Affiliations:** 1 Department of Neurobiology, Duke School of Medicine, Duke University, Durham, NC 27710; 2 Center for Cognitive Neuroscience, Duke University, Durham, NC 27708; 3 Department of Biostatistics & Bioinformatics, Duke School of Medicine, Duke University, Durham, NC 27710; 4 Department of Biomedical Engineering, Pratt School of Engineering, Duke University, Durham, NC 27708; 5 Department of Psychology & Neuroscience, Trinity College of Arts and Sciences, Duke University, Durham, NC 27708; 6 Duke Institute for Brain Sciences, Duke University, Durham, NC 27708

**Keywords:** active perception, Bayesian models, corollary discharge, primates, saccades, vision

## Abstract

The brain interprets sensory inputs to guide behavior, but behavior itself disrupts sensory inputs. Perceiving a coherent world while acting in it constitutes active perception. For example, saccadic eye movements displace visual images on the retina and yet the brain perceives visual stability. Because this percept of visual stability has been shown to be influenced by prior expectations, we tested the hypothesis that it is Bayesian. The key prediction was that priors would be used more as sensory uncertainty increases. Humans and rhesus macaques reported whether an image moved during saccades. We manipulated both prior expectations and levels of sensory uncertainty. All psychophysical data were compared with the predictions of Bayesian ideal observer models. We found that humans were Bayesian for continuous judgments. For categorical judgments, however, they were anti-Bayesian: they used their priors less with greater uncertainty. We studied this categorical result further in macaques. The animals’ judgments were similarly anti-Bayesian for sensory uncertainty caused by external, image noise, but Bayesian for uncertainty due to internal, motor-driven noise. A discriminative learning model explained the anti-Bayesian effects. We conclude that active vision uses both Bayesian and discriminative models depending on task requirements (continuous vs categorical) and the source of uncertainty (image noise vs motor-driven noise). In the context of previous knowledge about the saccadic system, our results provide an example of how the comparative analysis of Bayesian versus non-Bayesian models of perception offers novel insights into underlying neural organization.

## Significance Statement

Primate vision deals with two major sources of uncertainty: suppression from eye movements and noise in the environment. Fortunately, the brain also has prior knowledge about the body and the world. Systems that exploit such priors more to compensate for greater uncertainty are considered Bayesian. A major theme in neuroscience is that the brain is Bayesian. We tested that hypothesis for vision in the context of eye movements using an integrated computational-psychophysical approach. Bayesian models explained perception during movement-induced noise, but not environmental noise, for which a simpler, “discriminative” model sufficed. We conclude that primate vision is Bayesian to compensate for intrinsic, but not extrinsic, sources of uncertainty, an important distinction for designing and interpreting neural studies of perception.

## Introduction

Perception can be split into two theoretical stages. Sensory receptors encode physical stimuli into neural signals (Barlow, 1961) and provide evidence, E, for the stimulus, S, to the rest of the sensory system. The evidence is then decoded to infer the stimulus from the evidence (Johnson, 2000; Britten et al., 1996) and guide action. Under a probabilistic framework, the goal of decoding is to infer the probability of the stimulus given the evidence, P(S|E) (Murphy, 2013).

Models of decoding take two broad forms (Ng and Jordan, 2002). Discriminative models estimate P(S|E) directly; they draw boundaries between evidence states and map stimulus states onto them (Rumelhart et al., 1986; Hinton, 1992). Generative models, in contrast, build models of the world (von Helmholtz, 1924; Knill and Richards, 1996; R.P.N. Rao and Ballard, 1999). These include the joint probability of the stimulus and the evidence co-occurring, P(E, S). P(S|E) can be derived from the joint probability using Bayes’ rule. As such, Bayesian models are a common implementation of generative models. Although discriminative and Bayesian models can combine for perception (Gardner, 2019; DiCarlo et al., 2021; Sohn and Jazayeri, 2021), Bayesian models have been particularly influential in explaining how sensorimotor systems use prior knowledge to optimally resolve uncertainty (Jacobs, 1999; Ernst and Banks, 2002; Weiss et al., 2002; Knill and Saunders, 2003; Kording and Wolpert, 2004; Jazayeri and Shadlen, 2010; Girshick et al., 2011; Fetsch et al., 2012; Darlington et al., 2017).

Sensory uncertainty may be introduced at the input stage or arise from one’s own movements. Constructing a stable, predictable percept of the world while moving through it constitutes active perception (Gibson, 1966; Bajcsy, 1988). Active perception is fundamental for behavior and its dysfunction may contribute to psychiatric disorders (Feinberg and Guazzelli, 1999; Ford and Mathalon, 2005; Thakkar and Rolfs, 2019). An apt model system for studying active perception is visual processing across saccades in primates (Wurtz et al., 2011; Parr and Friston, 2017). Each saccade blurs and displaces the visual image on the retinas. To counter these disruptions, the primate visual system uses a copy of the saccade command, or “corollary discharge” to suppress the blur and nullify predicted displacements (Wurtz, 2018). At least part of this process, saccadic suppression of the blur, is the outcome of combining motor and sensory information across saccades in a Bayes optimal manner (Niemeier et al., 2003; Crevecoeur and Kording, 2017).

**Figure 1.**
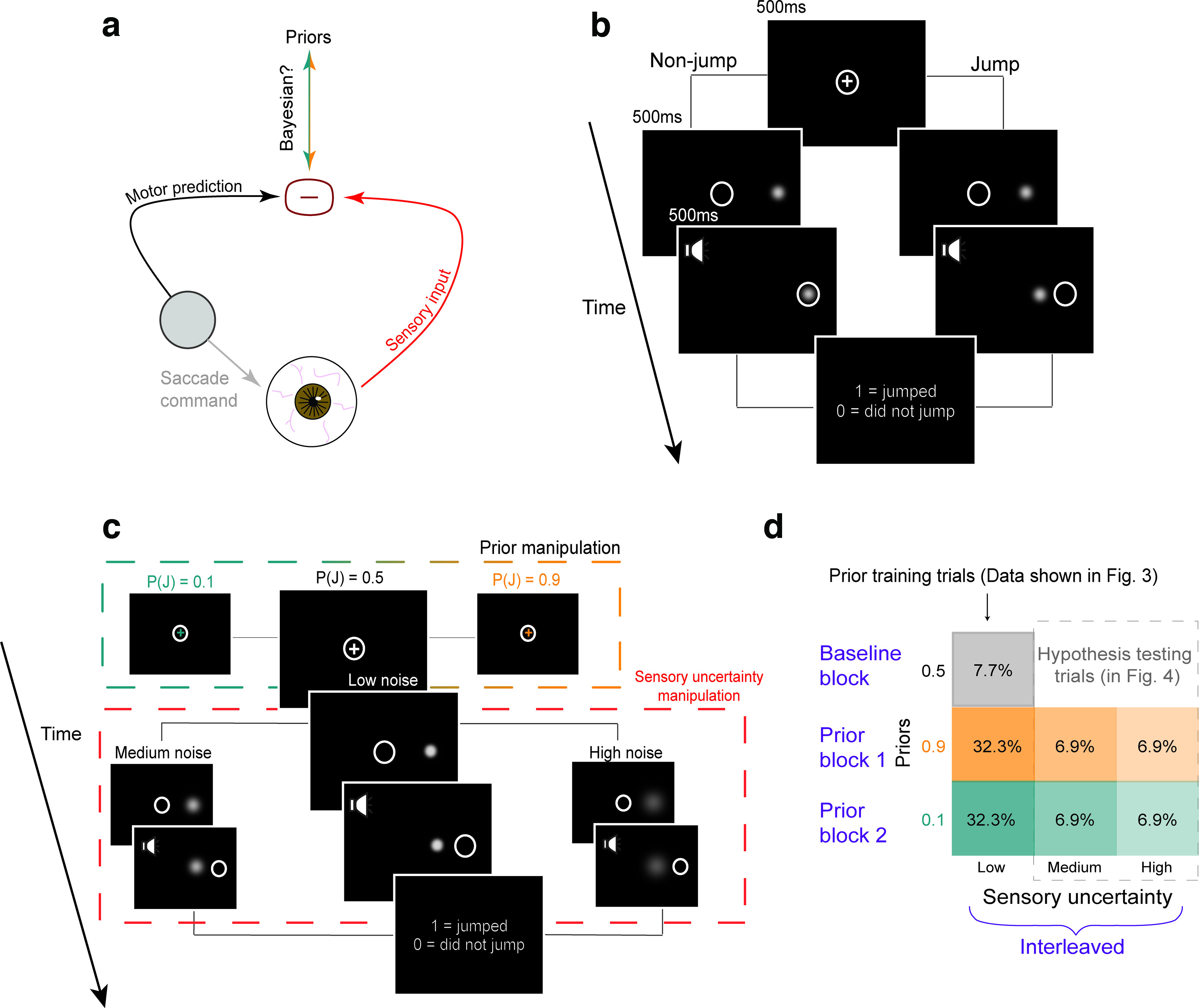
Experimental design. ***a***, Judging whether an object is stable or moves during a saccade involves comparing a motor-driven prediction with sensory input. We tested whether this process is Bayesian. ***b***, Schematic of the SSD task. Participants fixated on a central cross and on being cued, made a saccade to a peripheral target which either jumped or did not jump during the saccade. Participants reported whether they perceived it as having jumped or not. White circle: eye position. ***c***, Schematic of main experimental variables. Middle, larger panels, “Baseline” condition with neutral prior P(J) = 0.5 and low uncertainty (minimal blur). High (0.9) and low (0.1) priors were cued by the color of the fixation cross (top dashed box). Sensory noise was manipulated by the width of the Gaussian target (bottom dashed box). ***d***, Trial breakdown for Experiment 2. Numbers in the boxes indicate the overall proportion of each trial type. Blocks of high and low priors followed a baseline block (gray). 70% of trials in the prior blocks (i.e., in the orange or teal rows) were prior-training trials with low uncertainty and priors matched to true jump probability (results shown in Fig. 3). For each prior, training trials constituted 32.3% of all trials in the experiment. 30% were hypothesis testing trials with medium-uncertainty and high-uncertainty targets. Fixation colors cued the learned priors although the true jump probability was 0.5 (results shown in Fig. 4). Hypothesis testing trials formed 6.9% of all presented trials at each uncertainty level.

Here, we focused on whether Bayesian models are used to correct self-generated retinal displacements. The primate visual system, using corollary discharge, can predict its inputs after each saccade (Sommer and Wurtz, 2002, 2006; Vaziri et al., 2006) and compare that prediction with the postsaccadic visual input (Fig. 1*a*). A match means that a viewed object was stable. Previous work by H.M. Rao et al. (2016) showed that humans use priors about the probability of object movement for this process. In this study, we asked whether priors are used in a Bayesian manner, i.e., are they used more with greater sensory uncertainty?

First, we evaluated ways to induce sensory uncertainty (Experiment 1). Then we extended the paradigm of H.M. Rao et al. (2016) to test whether humans are Bayesian when reporting categorically if a stimulus moved or not during a saccade (Experiment 2). Surprisingly, participants were anti-Bayesian, using their priors less with increasing noise. Continuous judgments of target displacement, however, did yield Bayesian behavior (Experiment 3) as found previously in other systems. We studied the unexpected categorical task results further using macaques to allow for more precise eye movement monitoring, to achieve extensive within-subject testing, and to prepare for a study on the neural correlates of the behavior. We analyzed prior use separately for sensory uncertainty added to the external image (Experiment 4) or caused by self-movement (Experiment 5). The monkeys were anti-Bayesian for image noise, like the humans, but Bayesian in compensating for motor-induced noise. A Discriminative learning-based model provided a feasible explanation for the anti-Bayesian results.

## Materials and Methods

We have split Materials and Methods into two sections, “Experimental design and statistical analyses” and “Modeling”. The Experimental design and statistical analyses section has three subsections. The first subsection includes the methods for the psychophysics experiments run on humans. This includes an initial experiment to identify a sensory noise manipulation (Experiment 1), an experiment testing the trade-off between categorical priors and sensory uncertainty (Experiment 2), and an experiment testing the trade-off between continuous priors and sensory uncertainty (Experiment 3). The second subsection details the methods for the experiments run on rhesus macaques. This subsection includes experiments to isolate the trade-off between a categorical prior and visual uncertainty alone (Experiment 4) or motor uncertainty alone (Experiment 5), as well as a control experiment. The third subsection includes a description of the data preparation and analysis measures used throughout the manuscript. The Modeling section includes detailed descriptions of the Bayesian computational models used throughout the manuscript.

### Experimental design and statistical analyses

#### Human psychophysics

##### Materials and paradigm

Forty-five adult volunteers with normal or corrected-to-normal vision participated in the experiments. All procedures were explained verbally to participants beforehand and written, informed consent was obtained. Participants were paid $12/h and informed that participation was completely voluntary. All procedures were performed in accordance with protocols approved by the Duke University Institutional Review Board.

Participants sat alone in a darkened room in front of a monitor with their head stabilized using a chin-rest and forehead-rest. The monitor was positioned at 60 cm from the center of the head. Experiments 1 and 2 were displayed on a 21′′ CRT monitor (Accusync 120) at 120 Hz. Experiment 3 was displayed on a 24′′ Dell LCD monitor at 60 Hz. This places the edge of the monitor at 18° for Experiments 1 and 2, and 24° for Experiment 3. The experiment was written in and displayed using Presentation (Neurobehavioral Systems). Monocular eye position was recorded with an eye-tracking system developed by Matsuda et al. (2017).

Participants performed a modified Saccadic Suppression of Displacement (SSD) task (Bridgeman et al., 1975; Fig. 1*b*). On each trial, a fixation cross first appeared near the center of the screen. Once participants acquired and maintained fixation for 500 ms, a saccade target appeared at one of two average positions relative to the center of the screen: 10° or −10°. A target at 10° appeared in the right half of the screen, whereas a target at −10° appeared in the left half of the screen. Additionally, on every trial, the position of the target and fixation cross were both jittered by −0.5 to 0.5° relative to the average position to mitigate the confounding effects of adaptation to either a constant saccade amplitude or a constant distance between the target and the edge of the screen. The fixation cross then disappeared for 500 ms, and an auditory cue was presented to signal to participants that they were allowed to make a saccade to the target. If fixation was broken before the auditory cue, the trial was aborted and a new one began immediately. Saccade initiation (defined as the time the eye left a window of 2° relative to the fixation cross) triggered target displacement. In Experiments 1 and 2, participants provided a binary report on whether they had perceived the target as having moved or not. The target remained on the screen for 500 ms after it was displaced, after which it was replaced by a response prompt screen (5 = moved, 0 = remained stationary). In these experiments, target displacement was drawn from overlapping Gaussian distributions designated as the “movement” and “nonmovement” distributions. On trials where the target moved, the displacement was drawn from a relatively broad Gaussian distribution centered around 0 (μ = 0°, σ = 1.5°). On “no movement” trials, the displacement was drawn from a very narrow Gaussian distribution centered around 0 (μ = 0°, σ = 0.017°). A positive displacement meant that the target moved rightward, and a negative displacement meant it moved leftward.

In Experiment 3, participants provided a continuous report of the target’s postsaccadic location. For this study, the target stayed visible for 50 ms after displacement and was then replaced by a screen where the mouse cursor (shaped “+”) was placed at the center of the screen and restricted to the horizontal meridian. Participants could then move the mouse cursor to where they perceived the target as having landed.

##### Experiment 1: testing stimuli for the sensory uncertainty manipulation

Throughout the study we manipulated two sets of independent variables, priors and sensory noise. Our approach to manipulating priors was based on a previously established procedure (H.M. Rao et al., 2016). There are many potential ways to introduce sensory noise, however, and it was unclear which would be the best method for our goal of parametrically obscuring the detection of image movement. Experiment 1 evaluated several options to achieve this goal. Nine human participants completed at least 100 trials each in eight experimental conditions: four candidate noise-manipulation stimuli at two uncertainty levels each. The probability of target movement across all stimulus conditions was 0.5.

The four possible noise-manipulation stimuli were:

1. Arrow targets (1° long with 0.5° width) that pointed either in the direction of their movement (congruent) or in the opposite direction (incongruent). The prediction was that incongruent movements (opposite to the direction indicated by the arrow) would induce greater uncertainty and decrease discriminability.
2. Targets consisting of a Gaussian cloud of 20 white squares (0.25° × 0.25°) for which the uncertainty corresponded to the SD of the cloud (low uncertainty = 0.063° and high uncertainty = 0.25°).
3. Targets consisting of squares (0.5° × 0.5°) at two levels of contrast (low uncertainty = 0.78 and high uncertainty = 0.29).
4. Targets that were Gaussian “blobs” (Gaussian distributions of light) for which uncertainty corresponded to the SD of the blob (low uncertainty = 0.19° and high uncertainty = 0.47°).

The outcome of this experiment determined how we manipulated the sensory uncertainty in the rest of the experiments.

##### Experiment 2: trade-off between binary prior and sensory uncertainty in humans

We trained participants on the other independent variable, the prior, by cuing the probability of object movement by the color of the fixation cross and using performance-based feedback. They were told whether their responses were correct or incorrect on each trial using an image of a smiling or frowning face, respectively. Based on the results from Experiment 1, we chose the Gaussian blob as sensory uncertainty manipulation (Fig. 1*c*). The target was grayscale on every trial; only its width given by the SD changed. The target had one of three possible SDs for the whole experiment: 0.1° (“low noise”), 0.25° (“medium noise”), or 0.5° (“high noise”).

Twenty participants completed a total of 1300 trials each. Trials were presented in 100-trial blocks. For all participants, the first block was a baseline block where the color of the fixation cross was white, and the target moved on 50% of the trials. In the next six blocks, the fixation cross was either green or red, and vice versa for the last six. Each of these fixation colors was associated with one of two probabilities of target movement (0.9 or 0.1). The order of the two prior conditions and color-probability associations were counterbalanced across participants. As in Experiment 1, displacements were drawn from a relatively broad Gaussian distribution (μ = 0°, σ = 1.5°) on “movement” trials and from a narrow Gaussian distribution (μ = 0°, σ = 0.017°) on “nonmovement” trials to ensure that the solution to the task was probabilistic. Thus, the optimal solution to the task was to learn the probability that any given displacement was drawn from the “movement” distribution relative to the “nonmovement” distribution. In conditions with a biased prior (0.9 or 0.1), the optimal solution would be to weight this relative probability by the appropriate prior (detailed mathematical description in Materials and Methods, Modeling). In other words, the optimal solution to this task is the Bayesian solution.

For 70% of the trials in blocks 2–13, the target had the lowest noise (SD of 0.1°) and the probability of target displacement conformed to the experimental prior, i.e., 0.9 or 0.1. These 70% of the trials were considered “training trials” where the intended prior was reinforced and maintained. The other 30% of trials were “testing trials,” where we tested the hypothesis that participants would use their learned prior more when the evidence was relatively uncertain. On these trials, the target had either medium or high sensory noise. Additionally, both to isolate the effects of a learned, color-associated expectation on performance and to mitigate the possibility that our sensory manipulation affected participants’ representation of the prior, the testing trials comprised a neutral condition where the target had a 0.5 probability of moving, but the fixation color cuing the prior was the same as the rest of the block. Training and testing trials were randomly interleaved (Fig. 1*d*). To preserve a sense of experiential continuity across the experiment, 5% of the targets in block 1 had a SD of 0.25° (“medium noise”) and 5% had a SD of 0.5° (“high noise”). Data from these trials were not analyzed.

##### Experiment 3: trade-off between continuous prior and sensory uncertainty in human participants

Fourteen human volunteers participated in Experiment 3. We tested the hypothesis that the visual system uses Bayesian inference to determine the continuous displacement value of objects across saccades. The overall paradigm was similar to Experiments 1 and 2. The critical difference was that the target was displayed for a limited period (50 ms) after it moved and participants provided a continuous report of where they had perceived it as having landed. Participants fixated a central cross and on being cued, made a saccade to a target located at either 10° or −10°. The target was displaced horizontally during the saccade, displayed in its new location for 50 ms, and then replaced by a response screen. The response screen consisted of a mouse cursor (a white cross “+” that was 0.24° in size) that started out in the center and was restricted to the horizontal meridian to ensure that participants were solving a one-dimensional problem. Participants were required to drag the mouse cursor to the location where the target had landed and click to submit their response.

Participants completed a total of 1000 trials each. We first trained participants on the prior for 600 trials and then tested the use of this prior with increasing sensory noise. As in Experiments 1 and 2, the target was a Gaussian blob and we manipulated sensory uncertainty by varying its width. The prior was a continuous Gaussian distribution of displacements, rather than a categorical prior indicating the probability of object displacement. Throughout the experiment, displacements were drawn from a Gaussian distribution with mean 0° and SD 1°. Participants were trained on this prior in the first 600 trials with performance-based feedback. After they submitted their response, the target appeared in its correct postsaccadic location for 500 ms. To indicate their degree of correctness, the color of this feedback target ranged continuously from green (correct) to red (incorrect by >2°). Targets in this training phase trials had a SD of 0.1° (“low noise”).

In the remaining 400 trials, participants underwent a “testing” trials phase during which they were provided no feedback. These trials had one of three noise levels: 0.1° (“low noise”), 0.5° (“medium noise”), and 1° (“high noise”). Further, throughout the experiment, in 20% of the trials, the target did not appear postsaccadically. We call these “infinite-noise” trials. All four noise levels were randomly interleaved throughout the testing phase. All data shown come from the testing phase of the experiment. We used participants’ performance in the infinite-noise condition to evaluate how well they learned the prior in the training phase.

#### Rhesus macaque psychophysics

##### Materials and paradigm

Two rhesus macaques (Monkey S and Monkey T, both males) were trained to perform a modified Saccadic Suppression of Displacement paradigm, similar to the human participants. Animals were brought into the lab in custom-made chairs (Crist Instruments) and their heads were stabilized using a headpost that attached to both the chair and a surgically implanted socket (Crist Instruments) on the skull. The socket was implanted in an aseptic surgical procedure with the help of ceramic screws and acrylic. Eye position was measured using a surgically implanted scleral search coil (Robinson, 1963; Judge et al., 1980) in one eye. All surgical and experimental procedures were performed in accordance with protocols approved by the Duke Institutional Animal Care and Use Committee.

In a typical experimental session, the animals performed the behavioral task in a dark experimental rig. They were positioned 60 cm from an LCD monitor (1920 × 1080, 144 Hz). To dissociate external sources of sensory noise from internal, motor-driven sources, the saccade target was dissociated from a visual probe (a Gaussian blob) which was displaced intrasaccadically on some trials. In the human experiments, the Gaussian, visual probe (same as the saccade target) always appeared in one of two locations on the screen and only moved horizontally. For Experiments 4 and 5 in monkeys, it could appear in one of four locations, ±10° horizontally or ±10° vertically. The saccade target was always positioned along the orthogonal cardinal direction (e.g., if the probe appeared at ±10° horizontally, the saccade target would be at ±10° vertically), and the probe was displaced in a direction parallel to the saccade vector. For the control experiment, we simultaneously recorded from neurons while the animals performed the sessions (neural data not presented in this manuscript). Since we placed the probe within the mapped receptive field of the neuron, the probe appeared in a different location during each session.

On each trial, a fixation square (1° × 1°) first appeared at the center of the screen. After fixation had been acquired and maintained for a randomized duration of 300–500 ms, the visual probe appeared at one of the four locations on the screen for 500–700 ms. The monkey was required to maintain fixation on the central fixation square for that duration, after which the fixation square was replaced by the saccade target (1° × 1°) indicating to the animal they could make a saccade. Saccade initiation (defined as the time the eye crossed a threshold set at 20% of the saccade length, i.e., 2°, in the direction of the saccade) triggered the displacement of the probe on some trials. The probe was displaced in a direction parallel to the saccade. Animals were further required to maintain postsaccadic fixation for 700 ms after which the saccade target was replaced by a white cross in the same location. To report that the probe had moved during the saccade, the monkey was required to make a saccade to the probe within 500 ms and then fixate on it for 400 ms. To report that it had remained stationary during the saccade, the monkey had to remain fixated on the cross for 1000 ms. The precise timing of stimulus presentation was verified with a photodiode taped to the top left corner of the monitor, where a white square (invisible to the monkey) was flashed within the same frame as the measured stimulus.

Displacements were drawn from relatively broad and narrow Gaussian distributions in the movement (μ = 0°, σ = 2.5°) and nonmovement (μ = 0°, σ = 0.2°) conditions, respectively. Positive displacements were either rightward or upward, and negative displacements were leftward or downward. Priors were cued by the color of the fixation and target squares. For monkey S, green squares meant that the probe had a 0.2 probability of being displaced while magenta squares indicated a 0.8 probability of displacement. For monkey T, blue squares were associated with a 0.2 probability of displacement while orange squares were associated with a 0.8 probability of displacement. Animals were trained on priors over multiple sessions using performance-based feedback like human participants.

##### Experiment 4: trade-off between categorical priors and visually-driven sensory uncertainty

To measure performance as a function of external sensory uncertainty, the visual probe in Experiment 4 was a Gaussian “blob” with one of three possible SDs: 0.5° (“low noise”), 1.25° (“medium noise”), and 2° (“high noise”) for Monkey S and 0.5° (“low noise”), 1.25° (“medium noise”), and 1.75° (“high noise”) for Monkey T. The relative frequencies of all seven trial types (two priors × three noise levels + baseline) were the same as in the categorical experiment for humans (Experiment 2). Baseline trials with white squares and 0.5 probability of displacement all had “no noise” visual probes. In the 0.2 and 0.8 prior conditions, 70% of trials had no noise and conformed to the displacement probability indicated by the prior. The remaining 30% of trials with low and high noise comprised a neutral “test” condition with a veridical jump probability of 0.5. All seven trial types were randomly interleaved.

##### Control experiment with valid prior statistics for all noise levels

We also performed a control experiment, the purpose of which was to determine whether the anti-Bayesian results might be an artifact of adapting to the 0.5 probability of target movement in the medium-noise and high-noise conditions and ignoring the learned priors. In this experiment, the probability of movement matched the prior, e.g., 0.8 or 0.2, for all noise levels. Visual noise levels were manipulated the same way as in Experiment 4.

##### Experiment 5: trade-off between categorical priors and motor-driven sensory uncertainty

To measure performance as a function of internal, motor-driven sensory uncertainty, we added a condition to the experiment where monkeys did not make a saccade. The purpose was to eliminate a major form of saccade-driven sensory uncertainty, the saccadic suppression of visual signals. The monkeys remained fixated in the center while the Gaussian, visual probe was displaced. This no-saccade condition served as the “low motor noise” condition and was compared with a “high motor noise” condition where animals made a saccade. The temporal structure of the no-saccade trials was identical to the trials with a saccade. No-saccade trials were implemented by assigning the location of the “saccade target” to be the same as the fixation square. There were three prior conditions: 0.2, 0.5, and 0.8. Colors indicating the priors were the same as in Experiment 4. The visual probe had a SD of 0.5°, the lowest noise condition, for all trials. All six trial types (three priors × two noise levels) were randomly interleaved.

#### Data preparation and analysis measures

##### Data preparation

Data from individual trials were analyzed offline to confirm that the visual probe landed in its displaced location before the end of the saccade. The saccade end time was defined as the time at which the eye velocity dropped below 0.04°/ms. For human participants, the time at which the target jump command was sent was recorded for each trial. Trials with a recorded jump time >1 whole frame (8.33 ms for Experiments 1 and 2, and 16.7 ms for Experiment 3) before the detected end of the saccade were excluded from analysis. Participants for whom at least 90% of all trials did not meet this criterion were excluded from analyses entirely. No participants were excluded in Experiment 1, three participants were excluded from Experiment 2, and three participants were excluded from Experiment 3. For the macaque experiments, we used a photodiode to verify the exact timing of stimulus presentation. Note that the timestamp from the photodiode indicated the presentation of a white square at the top left of the screen and the monitor refreshes frames as a raster. We verified the maximum duration of a frame as being 7 ms from top left to bottom right using a second photodiode. Since the probe was presented at various locations on the screen, we set the most conservative criterion such that the photodiode timestamp had to be at least 7 ms before the detected end of the saccade. Individual trials that did not meet this criterion (<10% of trials across all sessions) in the macaque data were excluded. Displacement times, measured in the comparable Experiments 2 and 4 in humans and monkeys, respectively, were closer to the saccade end for monkeys (mean: −7.63 ms, SD: 5.07) than for humans (mean: −17.25 ms, SD: 1.80 ms).

##### Psychometric curves and prior use

All data were analyzed using MATLAB (MathWorks). For Experiments 1, 2, 4, and 5, psychometric curves were fit to binary responses using the four-parameter logistic regression model:

(1) $$\begin{array}{c} y=\max+\frac{\min-max}{1+{\left(\frac{x}{thresh}\right)}^{slope}} \end{array},$$

where x is the absolute value of the presented displacement, y is the value of the psychometric function, min is the minimum value of the function (i.e., y at x = 0), max is the maximum value, thresh is the inflection point, and slope is the slope of the psychometric function. min, max, thresh, and slope terms were fit to binary data by minimizing mean squared error.

For all of our main analyses, we used the intercept of the psychometric curve as a measure of prior use in these experiments for statistical tests and for comparison with the predictions of the categorical Bayesian model. We chose the intercept as a way of quantifying upward or downward shifts in psychometric curves, or the bias across prior conditions, both because it can be derived directly from the psychometric curve, and because it is a more reliable estimate of bias in our paradigm than the threshold. That is, given that displacements were drawn from overlapping Gaussian distributions centered on 0°, the density of participant responses was highest at the lower end of the curve, making the intercept the most reliable curve-fit parameter. For human participants and in the low-noise, prior training trials for macaques, displacements were drawn from continuous distributions. In these conditions, we used the value of min as the intercept. For the medium-noise and high-noise hypothesis testing trials in macaques, displacements were discretized. There was a displacement = 0 condition. In these conditions, the intercept is simply the proportion of “moved” responses in the displacement = 0 condition.

For comparison with the intercept measure, we repeated all the analyses of prior use using two alternative measures. The first one was the Criterion measure from Signal Detection Theory (Green and Swets, 1966). Criterion provides another measure of bias in responses (i.e., a translational shift in psychometric curves). It is given by:

(2) $$\begin{array}{c} C=-0.5\left(Z\left(hit\;rate\right)+Z\left(false\;alarm\;rate\right)\right) \end{array},$$

where the hit rate is the proportion of “jumped” responses on trials in which the probe truly moved, and the false alarm rate is the proportion of “jumped” responses on trials in which the probe did not move. A second alternative measure was used for the control experiment in monkeys. In that experiment, all displacements were discretized to allow for direct comparison of neural data (not shown in this report), rather than being pulled from a continuous distribution. Therefore, instead of using the Criterion measure as an alternative to the intercept measure, we simply used the raw difference in response rates across all displacements. The results using Criterion and the raw response rates replicated the findings using the intercept measure and are shown in Extended Data Figures 4-1, 4-2, 7-3, and 8-1.

For all statistical comparisons, the assumption of normality was first tested for each sample using either a Kolmogorov–Smirnov (KS) test or a Shapiro–Wilk test. If met, we then used a parametric comparison such as an ANOVA or a *t* test. Otherwise, the equivalent nonparametric test was used.

##### Saccadic endpoint errors and scatter

Saccadic endpoints were defined as the average horizontal and vertical eye positions in the 60 ms following the end of the saccade (i.e., the time at which the eye velocity dropped below 0.04°/ms). We calculated endpoint errors as the difference between the endpoint location and target location. We then measured the scatter of endpoint errors as the SD of the error distribution in each condition.

### Modeling

#### Categorical Bayesian ideal observer model simulations

The results in Experiments 2, 4, and 5 were compared with the performance of a Bayesian ideal observer model in the categorical task. This section provides a detailed mathematical description of the model. A brief overview of the model and its key equations are also discussed in Results, Categorical judgments of displacement are “anti”-Bayesian.

The ideal observer makes a probabilistic decision about binary variable, J, indicating whether the target jumped or not. ¬ J indicates that the target did not jump. Since the true displacement is experimentally drawn but not available to the observer, they make this decision given the perceived displacement, $\hat{x}$. The decision is based on the relative probabilities of the target having jumped or not jumped given the perceived displacement:

(3) $$\begin{array}{c} D\left(\hat{x}\right)=I\left\{P(J|\hat{x})>P(\neg J|\hat{x})\right\} \end{array},$$

where $D(\hat{x}$) is the decision given the perceived displacement, $\hat{x}$, and is determined by a binary indicator function, I. I = 0 (no jump) if the condition in braces is not met. Otherwise, I = 1 (jumped); $P(J|\hat{x})$ is the probability that the probe jumped given $\hat{x}$; $P(\neg J|\hat{x})$ is the probability that the probe did not jump given $\hat{x}$.

Using Bayes’ rule for the condition within braces in Equation 3:

(4) $$\begin{array}{c} D\left(\hat{x}\right)=I\left\{\frac{P(\hat{x}|J)P(J)}{P(\hat{x}|\neg J)(1-P(J))}>1\right\} \end{array}.$$

The simulated decision of the ideal observer, however, must be compared with the responses of participants. We do not have access to participants’ perceived displacement, but instead can only infer their decision given the true experimental displacement, x. We assume that the perceived displacement is a Gaussian random variable where the mean is the true displacement, and its variance given by the width of the blob on that trial:

(5) $$\begin{array}{c} \hat{x}∼N\left(x,\sigma_{t}^{2}\right) \end{array},$$

where σ*_t_* is the variance of the target.

The decision given the true displacement can thus be modeled as follows:

(6) $$\begin{array}{c} D\left(x\right)=\int^{}I\left\{\frac{P(\hat{x}|J)P(J)}{P(\hat{x}|\neg J)(P\left(\neg J\right))}>1\right\}P(\hat{x}\text{|}x\text{)}dx \end{array}.$$

That is, the decision value given the true displacement, D(x), is the integral of the perceived displacement distribution that falls above the point at which the indicator function, I, is non-zero.

Based on the distributions used in the experiment,

(7) $$\begin{array}{c} x|J∼N\left(0,\sigma_{J}^{2}\right) \end{array}$$

and

(8) $$\begin{array}{c} x|\neg J∼N\left(0,\sigma_{\neg J}^{2}\right) \end{array}.$$

Since $P(\hat{x}\text{|}x\text{)}$, P(x|J), and P(x|¬ J) are Gaussian distributions, we integrate over x such that,

(9) $$\begin{array}{c} \hat{x}|J∼N\left(\mu_{J},\sigma_{J}^{2}+\sigma_{t}^{2}\right) \end{array}$$

and

(10) $$\begin{array}{c} \hat{x}|\neg J∼N\left(\mu_{\neg J},\sigma_{\neg J}^{2}+\sigma_{t}^{2}\right) \end{array}.$$

Thus, the expression inside the indicator function in Equation 6, when replaced with the appropriate Gaussian probability density functions, equals

(11) $$\begin{array}{c} \frac{\frac{1}{\sqrt{(\sigma_{J}^{2}+\sigma_{t}^{2})2\pi }}e^{-\frac{1}{2}\left(\frac{{\left(\hat{x}-\mu_{J}\right)}^{2}}{\sigma_{J}^{2}+\sigma_{t}^{2}}\right)}P(J)}{\frac{1}{\sqrt{(\sigma_{\neg J}^{2}+\sigma_{t}^{2})2\pi }}e^{-\frac{1}{2}\left(\frac{{\left(\hat{x}-\mu_{\neg J}\right)}^{2}}{\sigma_{\neg J}^{2}+\sigma_{t}^{2}}\right)}(P\left(\neg J\right))}>1 \end{array}.$$

Taking the log on both sides provides the condition under which the indicator function is >0,

(12) $$\begin{array}{c} log\left(\frac{\frac{1}{\sqrt{(\sigma_{J}^{2}+\sigma_{t}^{2})2\pi }}e^{-\frac{1}{2}\left(\frac{{\left(\hat{x}-\mu_{J}\right)}^{2}}{\sigma_{J}^{2}+\sigma_{t}^{2}}\right)}P(J)}{\frac{1}{\sqrt{(\sigma_{\neg J}^{2}+\sigma_{t}^{2})2\pi }}e^{-\frac{1}{2}\left(\frac{{\left(\hat{x}-\mu_{\neg J}\right)}^{2}}{\sigma_{J}^{2}+\sigma_{t}^{2}}\right)}(P\left(\neg J\right))}\right)>\log\left(1\right)=0 \end{array}.$$

That is,

(13) $$\begin{array}{c} log\left(\frac{1}{\sqrt{(\sigma_{J}^{2}+\sigma_{t}^{2})2\pi }}e^{-\frac{1}{2}\left(\frac{{\left(\hat{x}-\mu_{J}\right)}^{2}}{\sigma_{J}^{2}+\sigma_{t}^{2}}\right)}P(J)\right)-log\left(\frac{1}{\sqrt{(\sigma_{\neg J}^{2}+\sigma_{t}^{2})2\pi }}e^{-\frac{1}{2}\left(\frac{{\left(\hat{x}-\mu_{\neg J}\right)}^{2}}{\sigma_{J}^{2}+\sigma_{t}^{2}}\right)}P\left(\neg J\right)\right)>0 \end{array}.$$

Rearranging terms, the indicator function is >0 when

(14) $$\begin{array}{c} \frac{-{\left(\hat{x}-\mu_{J}\right)}^{2}}{\sigma_{J}^{2}+\sigma_{t}^{2}}+\frac{{\left(\hat{x}-\mu_{\neg J}\right)}^{2}}{\sigma_{\neg J}^{2}+\sigma_{t}^{2}}>log\left(\frac{\sigma_{J}^{2}+\sigma_{t}^{2}}{\sigma_{\neg J}^{2}+\sigma_{t}^{2}}\right)+2\log\frac{P\left(\neg J\right)}{P\left(J\right)} \end{array}.$$

When $\mu_{J}=\mu_{\neg J}=0$, the indicator function is >0 when ${\hat{x}}^{2}$ is greater than a criterion value, ${\hat{x}}_{C}^{2}$, defined as follows:

(15) $$\begin{array}{c} {\hat{x}}^{2}>{\hat{x}}_{C}^{2}=\frac{\text{log}\left(\frac{\sigma_{J}^{2}+\sigma_{t}^{2}}{\sigma_{\neg J}^{2}+\sigma_{t}^{2}}\right)+2\log\frac{P\left(\neg J\right)}{P\left(J\right)}}{\frac{1}{\sigma_{\neg J}^{2}+\sigma_{t}^{2}}-\frac{1}{\sigma_{J}^{2}+\sigma_{t}^{2}}} \end{array}.$$

When $\mu_{J}$ and $\mu_{\neg J}$ were non-zero, we identified the criterion by solving Equation 14 for $\hat{x}$ using MATLAB’s equation solver or the solve function.

Since $\hat{x}$ is a Gaussian random variable, $\hat{x}∼N(x,\sigma_{t}^{2}$), $\frac{{\hat{x}}^{2}}{\sigma_{t}^{2}}$ is a noncentral χ^2^ random variable, $\frac{{\hat{x}}^{2}}{\sigma_{t}^{2}}∼\chi^{2}\left(1,\frac{x^{2}}{\sigma_{t}^{2}}\right).$ Thus, the decision, D(x), can be modeled as the integral of a noncentral χ^2^ distribution that lies above the criterion, ${\hat{x}}_{C}^{2}.$ That is,

(16) $$\begin{array}{c} D\left(x\right)=1-F_{\chi^{2}}(\frac{{\hat{x}}_{C}^{2}}{\sigma_{t}^{2}},df=1,\lambda =\frac{x^{2}}{\sigma_{t}^{2}}) \end{array},$$

where $F_{\chi^{2}}$ is the cumulative distribution function of $\chi^{2}$ with degrees of freedom, df=1 and γ, $\lambda =\frac{x^{2}}{\sigma_{t}^{2}}$ up to $\frac{{\hat{x}}_{C}^{2}}{\sigma_{t}^{2}}$.

#### Categorical Bayesian ideal observer model fitting

We fit the model to data using maximum likelihood estimation, i.e., by minimizing negative log likelihood between the model’s output and subjects’ responses. While fitting, we assumed that subjects sometimes have lapses such that their psychometric curves do not always range from 0 to 1. To account for this, we allowed for two additional parameters, a lower bound and a lapse rate (1 – ceiling of the curve), and scaled the final output of the model as follows:

(17) $$\begin{array}{c} D\left(x\right)=lower\;bound+\left(1-lower\;bound-lapse\right)\times [1–F_{\chi^{2}}\left(\frac{{\hat{x}}_{C}^{2}}{\sigma_{t}^{2}},df=1,\lambda =\frac{x^{2}}{\sigma_{t}^{2}}\right)] \end{array}.$$

When fitting the model to data in Experiments 2 and 4, we allowed 14 parameters to be free: three priors, three noise levels, the widths of the “jump” and “nonjump” distributions, and a lower bound and lapse rate for each of the three noise levels.

#### Continuous Bayesian model: simulation and fitting

Results in Experiment 3 are compared with the performance of a Bayesian ideal observer for the continuous task. The ideal observer infers the perceived displacement as a reliability-weighted combination of the sensory likelihood and prior distributions,

(18) $$\begin{array}{c} D_{perceived}=w_{prior}D_{prior}+w_{likelihood}D_{likelihood} \end{array},$$

where $D_{perceived}$ is the mean of the inferred posterior distribution, $w_{prior}$ is the weight assigned to the prior, $D_{prior}$ is the mean of the prior distribution, $w_{likelihood}$ is the weight assigned to the likelihood, and $D_{likelihood}$ the likelihood distribution. When both $D_{prior}$ and $D_{likelihood}$ are Gaussian distributions, the weight terms are given by:

(19) $$\begin{array}{c} w_{prior}=\frac{\sigma_{likelihood}^{2}}{\sigma_{likelihood}^{2}+\sigma_{prior}^{2}} \end{array}$$

and

(20) $$\begin{array}{c} w_{likelihood}=\frac{\sigma_{prior}^{2}}{\sigma_{likelihood}^{2}+\sigma_{prior}^{2}} \end{array}.$$

That is, the more reliable (i.e., less variable) estimate is weighted higher. This reliability-weighted inference is additionally the Bayes optimal estimate because the variance of the estimate, $\sigma_{perceived}^{2},$ is lower than the variance of both the prior and the likelihood distributions:

(21) $$\begin{array}{c} \sigma_{perceived}^{2}=\frac{1}{\frac{1}{\sigma_{likelihood}^{2}}+\frac{1}{\sigma_{prior}^{2}}} \end{array}.$$

We simulated the final response as the mean of the posterior distribution, i.e., its maximum value. The values of the parameters used to simulate the ideal observer responses shown in Figure 5*c* were the same as the ones used in the experiment. To fit the model to data, we minimized squared error between each participant’s responses and those of the Bayesian ideal observer model to identify the best-fit values for their internal prior and likelihood distributions. Best-fit parameters were identified on a participant-by-participant basis. Parameter optimization was performed using MATLAB’s *fmincon* function.

### Code accessibility

All the code used for this report was generated with MATLAB using a Windows 10 PC. The code and all the data are available on GitHub at https://github.com/dsub-neuro/non-bayesian-behavior-paper (downloadable in a single ZIP file and citable as DOI https://zenodo.org/badge/latestdoi/541753447).

## Results

### Gaussian blurring induces uncertainty of image movement

The premise of the study was that, if active visual perception were Bayesian, subjects would use priors more when sensory uncertainty increased. That is, the visual system would rely more on prior expectations about image movement if it were harder to detect the movement, in accordance with Bayesian ideal observer modeling. To train priors or cue learned priors, we used the color of the fixation cross (Fig. 1*b*, top dashed box), similar to the method of H.M. Rao et al. (2016) in which the color of the target itself indicated the prior. Sensory uncertainty manipulations were a new addition to the paradigm, however, so to select a satisfactory approach we compared four potential methods to make image movement harder to detect, each at two levels of increasing uncertainty (Experiment 1; *n* = 9 human participants). The four methods were to use (1) an arrow image that jumped either “congruently” in the direction in which it pointed, thus having low sensory uncertainty, or incongruently, thus having high uncertainty; (2) a Gaussian cloud image of white squares in which the noise corresponded to the SD of the cloud (low noise = 0.0625° and high noise = 0.25°); (3) square images at two contrast levels (low noise = 78.4% and high noise = 29.4%); and (4) Gaussian “blob” images in which the noise corresponded to the SD of the blob (low noise = 0.19° and high noise = 0.47°).

**Figure 2.**
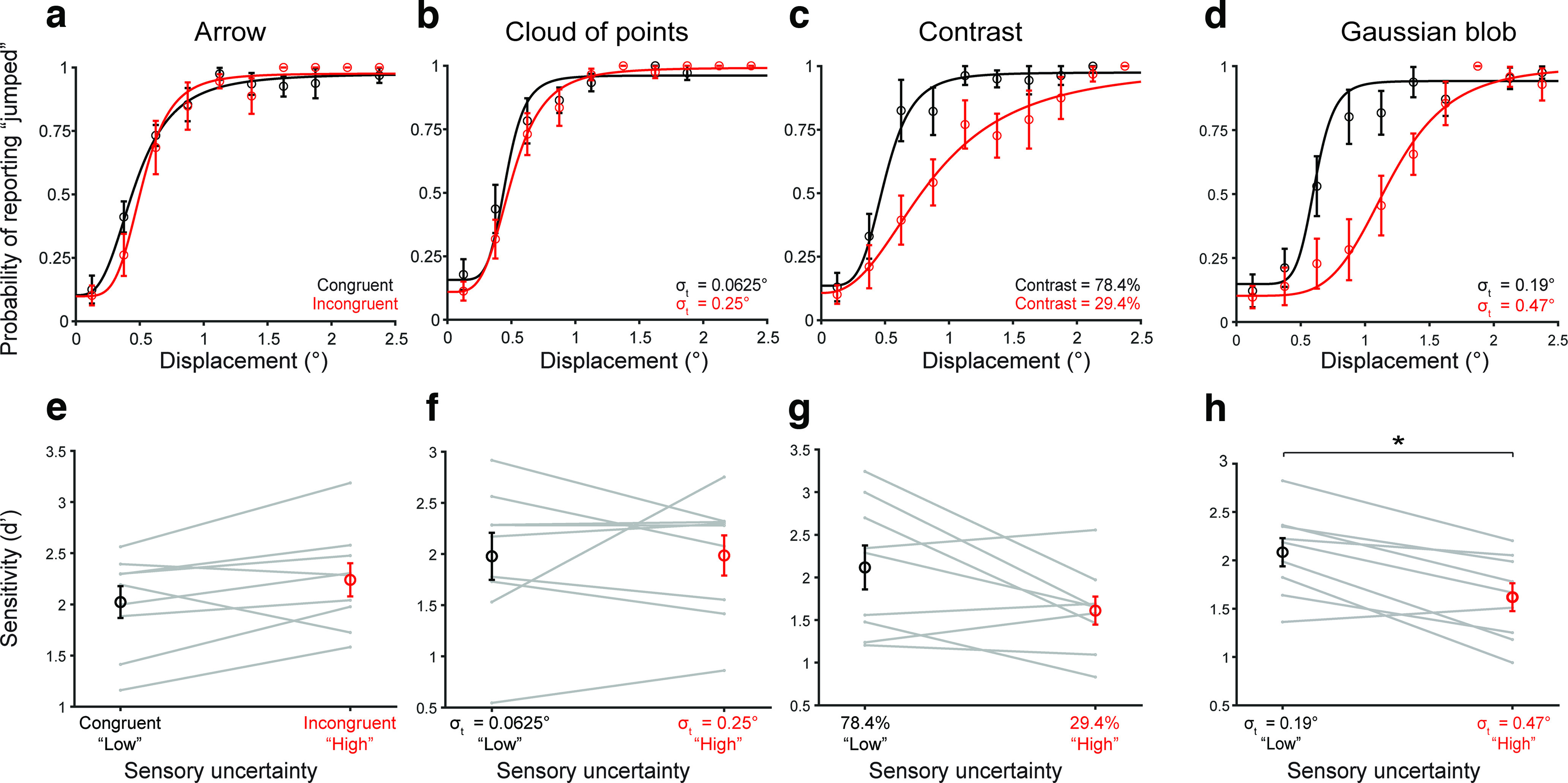
Evaluation of methods for manipulating image noise. Top row shows psychometric curves in the low (black) and high (red) noise conditions. Bins averaged across participants. Error bars: SEM. Curves were fit to pooled data. Bottom row shows d’ values in the two noise conditions. Gray lines: individual participants. Markers and error bars: means and SEM across participants. ***a***, ***e***, Congruent and incongruent arrow stimulus. ***b***, ***f***, Gaussian cloud of points. ***c***, ***g***, High and low contrast stimuli. ***d***, ***h***, Gaussian blob stimulus (emphasized by a gray box since it is the manipulation we selected to use for the rest of the experiments). *p < 0.0125.

For the arrow (Fig. 2*a*,*e*) and Gaussian cloud (Fig. 2*b*,*f*) manipulations, psychometric curves (fit to pooled data across participants) did not change in steepness between the low (black) and high noise (red) conditions. We also found no significant difference in sensitivity, measured by d’ (Green and Swets, 1966), between the low noise (mean = 2.02, SE = 0.16 for arrow; mean = 1.98, SE = 0.23 for Gaussian cloud) and high noise (mean = 2.24, SE = 0.16 for arrow; mean = 1.99, SE = 0.20 for Gaussian cloud) conditions (*p* = 0.091 and *p* = 0.97 for Gaussian cloud on a paired *t* test).

For stimuli with different contrasts (Fig. 2*c*,*g*), sensitivity in the low noise condition (mean = 2.12, SE = 0.16) trended higher than in the high noise condition (mean = 1.61, SE = 0.16), but the difference was not statistically significant at *n* = 9 participants (*p* = 0.055 on a paired *t* test). However, increasing the SD of a Gaussian “blob” target (Fig. 2*d*,*h*, highlighted with a box) reliably induced sensory uncertainty. This manipulation yielded psychometric functions that were steeper in the lower-noise condition (Fig. 2*d*, black curve, σ_t_ = 0.19°) than in the high-noise condition (Fig. 2*d*, red curve, σ_t_ = 0.47°). There were significant differences in sensitivity to target jumps, measured by d’ (Green and Swets, 1966), between the low-noise (mean = 2.08, SE = 0.15) and high-noise (mean = 1.62, SE = 0.14) levels (*p* = 0.0023 on a paired *t* test, Bonferroni corrected for four comparisons; Fig. 2*h*). Therefore, we chose Gaussian blobs of varying widths as the targets for the remaining experiments.

### Categorical judgments of displacement are “anti”-Bayesian

In Experiment 2, we used a modified Saccadic Suppression of Displacement (SSD) task (Bridgeman et al., 1975) in which we manipulated two sets of independent variables, priors and sensory noise (Fig. 1*c*), to test the Bayesian hypothesis. Human participants fixated near the center of a screen, and on being cued, made a saccade to a target. During the saccade, the target was displaced by varying amounts. After the saccade, participants reported their perception of whether the target had moved or not. Sensory uncertainty was induced by using Gaussian blob targets having widths (σ_t_) of σ_t_ = 0.1° (low noise), σ_t_ = 0.25° (medium noise), or σ_t_ = 0.5° (high noise). Participants were trained on priors, P(J), using performance-based feedback. Prior training trials constituted 70% of trials in each prior block (Fig. 1*d*). The fixation color indicated P(J) = 0.1 or 0.9 and the target had the lowest uncertainty, essentially punctate. Participants’ use of the prior, an independent variable, would be indicated by an increased probability of reporting “jumped” in the high prior condition and a decreased probability of doing so in the low prior condition, both relative to the baseline condition.

The remaining 30% of trials were “hypothesis testing” trials. The color of the fixation cross indicated the prior, but the true jump probability was a neutral 0.5 to isolate the effects of the learned, color-cued priors. Targets in these trials had additional sensory uncertainty (“medium” or “high”). The purpose of these trials was to measure the key dependent variable, the interaction of priors with sensory noise, to test the key Bayesian hypothesis that prior use increases with increasing sensory noise. The hypothesis testing trials were relatively infrequent and interspersed randomly to mitigate the possibility of participants recognizing that higher noise targets implied a neutral prior. We also performed a control experiment in which the jump probability matched the priors across noise conditions, as described below.

**Figure 3.**
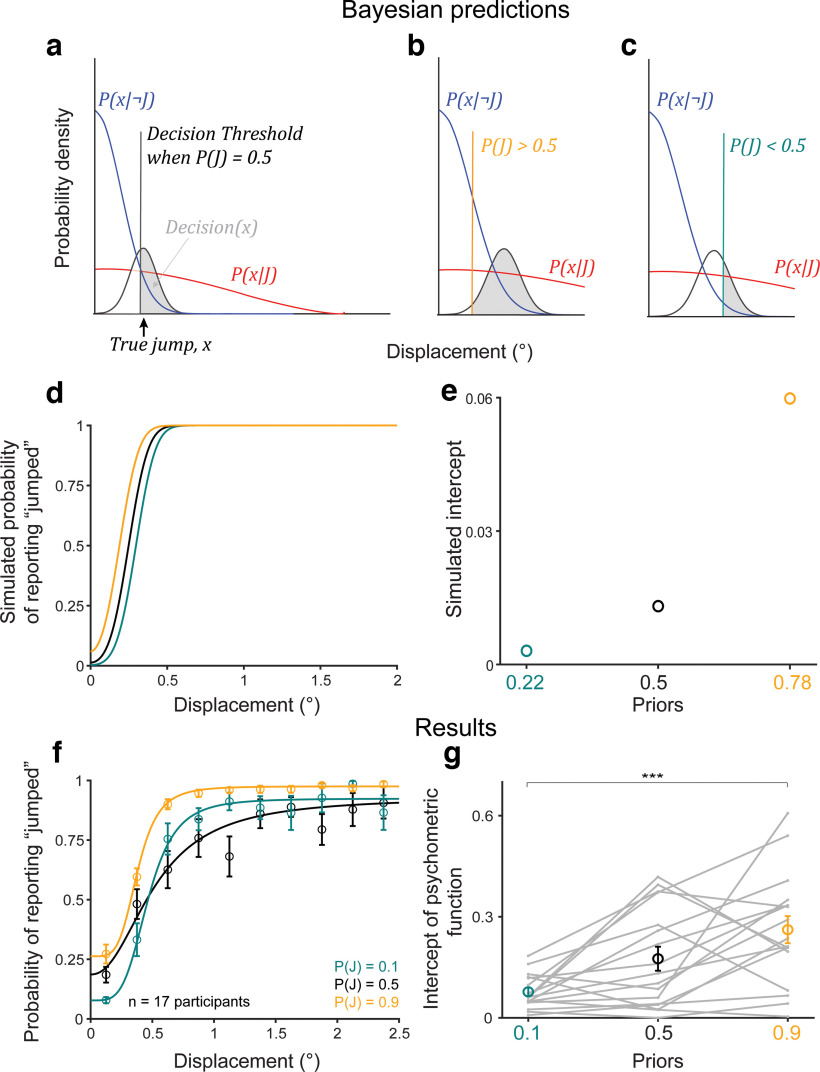
Participants learned the priors. ***a–c***, Bayesian ideal observer models for the three prior conditions. ***d***, Bayesian predictions for prior learning. ***e***, Intercepts for curves in ***d***. ***f***, Psychometric curves from *n* = 17 participants. Psychometric curves split by the direction of the displacement relative to the saccade direction are shown in Extended Data Figure 3-1. ***g***, Intercepts for curves in ***f***, fit to individual participants, matched Bayesian predictions in ***e***. ***p < 0.001.

To analyze data in Experiment 2, we compared participants’ performance with the predictions of a Bayesian ideal observer model (Fig. 3*a–c*; for details, see Materials and Methods, Modeling). On every trial, the ideal observer decided whether the probe jumped or not given a perceived displacement, $\hat{x}$. The decision would be “yes” if the probability of a jump given $\hat{x}$, $P(J|\hat{x}),$ exceeded the probability of nonjump given $\hat{x}$, $P(\neg J|\hat{x})$:

(22) $$\begin{array}{c} D\left(\hat{x}\right)=I\left\{P(J|\hat{x})>P(\neg J|\hat{x})\right\} \end{array}.$$

**Figure 4.**
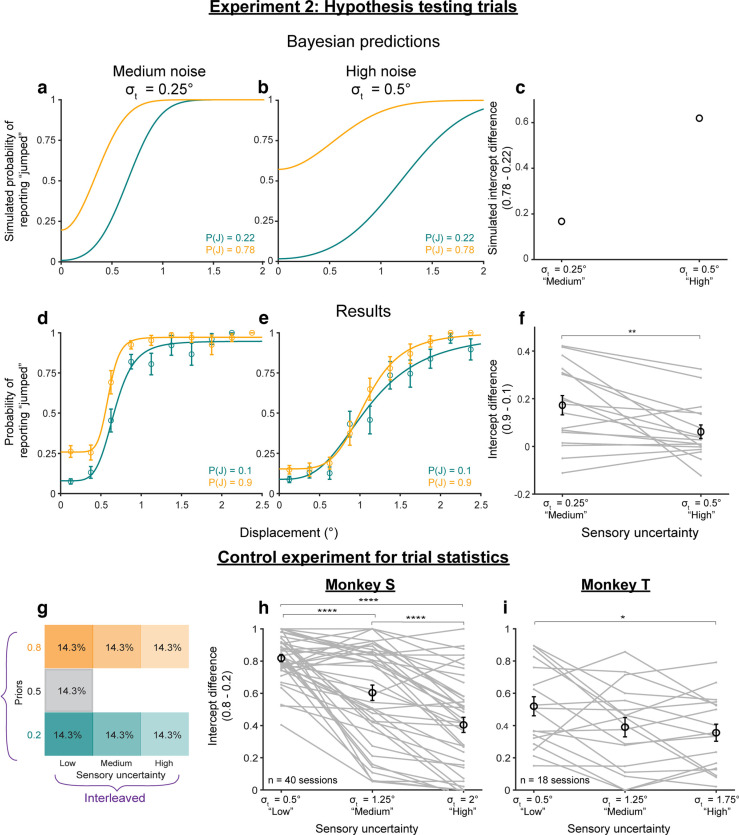
Categorical judgments of displacement are anti-Bayesian. ***a***, ***b***, Predicted psychometric curves from the Bayesian ideal observer model for the (***a***) medium-noise and (***b***) high-noise conditions. ***c***, High-low prior intercept differences for the curves in ***a***, ***b***. ***d–f***, Results from *n* = 17 participants for the (***d***) medium-noise and (***e***) high-noise conditions, and (***f***) the respective high-low prior intercept differences. **p < 0.01. Extended Data Figure 4-1 shows that the results replicate using Criterion as a measure of prior use. ***g***, ***h***, Results from a control experiment run on monkeys, in which the true jump probability matched the prior for the medium-noise and high-noise trials. ***g***, Trial breakdown. ***h***, ***i***, High-low prior intercept difference across noise levels for (***h***) Monkey S and (***i***) Monkey T. ****p < 0.0001, *p < 0.05. Extended Data Figure 4-2 shows results when the difference in the “jump” response rates for all displacements, rather than just the intercepts, were used as a measure of prior use. Extended Data Figure 4-3 shows that the results in the control experiment did not change between sessions in the first and second halves of the experiment. Extended Data Figure 4-4 shows the results of fitting the Bayesian ideal observer model to the data in Experiment 2.

10.1523/ENEURO.0403-22.2023.f3-1 Extended Data Figure 3-1 The direction of target displacement relative to the saccade did not influence the results of Experiment 2. Top, Data from displacements in the direction of the saccade. Bottom, Data from displacements opposite to the direction of the saccade. ***a***, ***d***, Data from prior learning trials. Participants demonstrated that they learned the prior regardless of the direction of the displacement (compare to pooled data in Fig. 3*f*). ***b***, ***e***, Data from hypothesis-testing, medium-noise trials. ***c***, ***f***, Data from hypothesis-testing, high-noise trials. As with the pooled data (Fig. 4*d*,*e*), prior use decreased with increasing noise for both displacement directions. Plotting conventions are the same as in Figures 3 and 4. Overall, the direction of target displacement relative to the saccade did not matter, as seen by comparing the data shown here with the pooled data of Figures 3 and 4. Download Figure 3-1, TIF file.

10.1523/ENEURO.0403-22.2023.f4-1 Extended Data Figure 4-1 Replication of the Experiment 2 results using Criterion instead of intercepts. ***a***, Criterion decreased with the prior magnitude, demonstrating that the human participants learned the priors in training trials. Note that a lower Criterion value meant that participants were more likely to report “jumped.” Plotting conventions as in Figure 3*g*. *F*_(2)_ = 22.20, *p* = 8.97 × 10^−7^ on a repeated-measures ANOVA. *Post hoc* comparisons using a Tukey’s HSD test showed that high prior Criterion values (−0.11 ± 0.10) was significantly lower than baseline (0.30 ± 0.08; *p* = 0.00028) and low prior (0.48 ± 0.06; *p* = 7.16 × 10^−7^) values. Low prior and baseline values were not significantly different from each other (*p* = 0.11137). ***b***, The difference in Criterion values between low and high priors was higher in the medium-noise condition (0.54 ± 0.13) than in the high-noise condition (0.21 ± 0.12; *p* = 0.0055 on a paired *t* test). In other words, prior use as measured by Criterion differences decreased as sensory uncertainty increased, the same result as when using intercept differences (compare Fig. 4*e*). Plotting conventions as in Figure 4*e*. Download Figure 4-1, TIF file.

10.1523/ENEURO.0403-22.2023.f4-2 Extended Data Figure 4-2 Replication of the control experiment results using response rates for all displacements instead of intercepts. For both monkeys, S (***a***) and T (***b***), the difference in response rates decreased as sensory uncertainty increased (*p* = 1.57 × 10^−10^ on a Friedman test for Monkey S, and *p* = 0.0014 on a repeated-measures ANOVA for Monkey T). *Post hoc* comparisons using pairwise signed-rank exact tests showed that prior use in the high-noise condition (0.40 ± 0.04) was significantly lower than in the low (0.79 ± 0.02; *p* = 6 × 10^−11^) and medium-noise (0.57 ± 0.04; *p* = 2.28 × 10^−7^) conditions, and prior use was significantly different in the low-noise and medium-noise conditions (*p* = 8.85 × 10^−6^) for Monkey S. For Monkey T, *post hoc* comparisons using Tukey’s HSD tests showed that prior use in the low-noise (0.55 ± 0.05) condition was significantly higher than in the medium-noise (0.39 ± 0.05; *p* = 0.0030) and high-noise (0.40 ± 0.05; *p* = 0.0056) conditions. In summary, they used their priors less with greater image noise, the same result as when using intercept differences (compare Fig. 4*h*,*i*). Plotting conventions as in Figure 4*h*,*i*. Download Figure 4-2, TIF file.

10.1523/ENEURO.0403-22.2023.f4-3 Extended Data Figure 4-3 Prior use decreased with increasing sensory noise within each chronological half of the control experiment. ***a***, ***b***, Results for Monkey S (***a***, *p* = 2.28 × 10^−9^ on a repeated-measures ANOVA in the first half; ***b***, *p* = 1.43 × 10^−6^ on a Friedman test in the second half). ***c***, ***d***, Results for Monkey T [***c***, *p* = 0.033 in the first half; ***d***, *p* = 0.18 in the second half (i.e., decreasing but not significant) on repeated-measures ANOVAs]. For Monkey S in the first half, *post hoc* Tukey’s HSD comparisons for Monkey S showed that the intercept difference in the low-noise condition (0.75 ± 0.03) was significantly higher than in the medium-noise (0.56 ± 0.07; *p* = 0.0019) and high-noise (0.35 ± 0.06; *p* = 2.28 × 10^−5^) conditions, and in the medium-noise condition was significantly higher than in the high-noise condition (*p* = 0.00025). In the second half (paired signed-rank exact tests), the intercept difference in the low-noise condition (0.89 ± 0.02) was significantly higher than in the medium-noise (0.64 ± 0.07; *p* = 0.0031) and high-noise (0.46 ± 0.08; *p* = 9.57 × 10^−5^) conditions, and in the medium-noise condition was significantly higher than in the high-noise condition (*p* = 0.0014). For Monkey T in the first half, the intercept difference in the low-noise condition (0.43 ± 0.07) was significantly higher than in the medium-noise (0.27 ± 0.06; *p* = 0.04) condition, but not between the other conditions. Download Figure 4-3, TIF file.

10.1523/ENEURO.0403-22.2023.f4-4 Extended Data Figure 4-4 Bayesian ideal observer model fits in Experiment 2 did not recapitulate the patterns observed in the data. ***a–c***, The model recapitulated the observed patterns in the binned, empirical data reasonably well for the low-noise (***a***), medium-noise (***b***) conditions. However, for the high-noise condition (***c***), it systematically overestimated the probability of reporting “jumped” in the high prior condition, and therefore, prior use. ***d***, ***e***, Fit prior (***d***) and sensory noise (***e***) parameter values. ***f***, Histogram of objective function (i.e., model error) values for all participants. ***g–i***, Same as ***a–c***, but for the four participants with the lowest model error values (<300). The same overall pattern of deviation from the data was observed, i.e., the model overestimated prior use in the high-noise condition. Fit lapse rates in the highest noise condition were higher than in the other two conditions (low noise: 0.04 ± 0.009, medium noise: 0.03 ± 0.01, high noise: 0.17 ± 0.05), and the widths of the “jump” distribution (3.03 ± 0.35) were higher than those of the “nonjump” distribution (0.47 ± 0.17) as expected. Download Figure 4-4, TIF file.

The decision variable $D(\hat{x}$) is determined by a binary indicator function, I. I = 0 (no jump) if the condition in braces is not met. Otherwise, I = 1 (jumped). Using Bayes’ rule,

(23) $$\begin{array}{c} D\left(\hat{x}\right)=I\left\{P(\hat{x}|J)P(J)>P(\hat{x}|\neg J)(1-P(J))\right\} \end{array},$$

where $P(\hat{x}|J)$ and $P(\hat{x}|\neg J)$ are the likelihoods of “jump” and “no jump,” respectively. For each prior, P(J), there was a threshold at which the condition in braces was met, i.e., it was equally likely that the probe jumped or did not. For P(J) = 0.5, it was where the two likelihood distributions intersected and were equal (Fig. 3*a*, black vertical line). If there was no sensory uncertainty, i.e., if $\hat{x}$ = x where x was the true displacement, the ideal observer would report “no jump” for all displacements less than the threshold and “jump” for all displacements above the threshold. Since the target was a Gaussian blob, we assume Gaussian uncertainty σ *_t_*, determined by the target, about the true displacement, x (Fig. 3*a*, black distribution):

(24) $$\begin{array}{c} \hat{x}∼N\left(x,\sigma_{t}\right) \end{array}.$$

Therefore, the decision given the true displacement, D(x), was the integral over values of x greater than the decision threshold (Fig. 3*a*, shaded region):

(25) $$\begin{array}{c} D\left(x\right)=\int^{}I\left\{\frac{P(\hat{x}|J)P(J)}{P(\hat{x}|\neg J)(P\left(\neg J\right))}>1\right\}P(\hat{x}\text{|}x\text{)}dx \end{array}.$$

This restricted the value of the decision to range from 0 to 1. One concern was that the participants might exhibit a perceptual bias either in the direction of the saccade, or opposite to the direction of the saccade, that might skew the uncertainty about the displacement (Honda, 1989; Ross et al., 1997; Morrone et al., 2005). To test this, we measured their accuracy in the task for both directions relative to the saccade. We used the baseline condition (neutral prior, lowest noise condition) for this analysis. We found no significant difference in accuracy (in saccade direction: 0.7526 ± 0.03, opposite to saccade direction: 0.7499 ± 0.03, *p* = 0.9140; paired *t* test).

We first assessed prior learning, one of our key independent variables in the task. For a high prior, e.g., P(J) = 0.9, the threshold would move to the left since $P(\hat{x}|J)$ was weighted higher than $P(\hat{x}|\neg J)$, thus increasing the ratio in the braces (Fig. 3*b*), and vice versa for a lower prior, e.g., P(J) = 0.1 (Fig. 3*c*). Critically, for the same perceived displacement, the ideal observer was more likely to report that the probe jumped for a higher prior than for a lower prior. Figure 3*d* shows simulations for an ideal observer with likelihood distributions, $P(\hat{x}|J)∼N(0,2)$ and $P(\hat{x}|\neg J)∼N(0,0.017)$, prior P(J) = 0.22 (teal), P(J) = 0.5 (black), and P(J) = 0.78 (orange), and sensory noise, $\hat{x}∼\text{N}(x,0.1$). We chose P(J) = 0.22 and 0.78 for the simulations to account for 70% true-statistic trials and 30% neutral. The key point was that the high prior was >0.5 and the low prior was <0.5. Figure 3*e* shows the value of the curves at displacement = 0 (the intercept).

For the human participants (*n* = 17; Fig. 3*f*), in the prior training trials, psychometric curves shifted upward for the high prior (orange curve) and downward for the low prior (teal curve) at small displacements as predicted. We confirmed that the shift in psychometric curves was the same both in the direction of the saccade and opposite to the saccade before pooling data across the two conditions (Extended Data Fig. 3-1*a*,*d*). The crossing of the low-prior (teal) curve over the black was not predicted but has implications that are addressed below in Results, A discriminative model provides a candidate explanation for anti-Bayesian categorization. A lower intercept in the low prior condition than in the high prior condition (Fig. 3*g*) matched the Bayesian predictions in Figure 3*e*. Repeated-measures ANOVA on the intercepts with prior as the within-conditions factor yielded a significant main effect of priors (*F*_(2)_ = 11.82; *p* = 0.0001). *Post hoc* comparison (Tukey’s HSD) of the P(J) = 0.9 and 0.1 conditions, the two priors tested later in hypothesis testing trials, showed that high-prior intercepts (mean = 0.26, SE = 0.04) were significantly higher than low-prior intercepts (mean = 0.08, SE = 0.01; *p* = 2.79 × 10^−4^). These results indicated that participants learned the priors as expected, allowing us to evaluate the critical dependent variable: the change in prior use with increased sensory uncertainty.

In the randomized, less frequent hypothesis testing trials, we tested the Bayesian hypothesis that priors are used more with increasing uncertainty. In these trials, the targets had medium or high sensory uncertainty. Figure 4*a*,*b* shows Bayesian predictions for these medium-noise and high-noise conditions, respectively. We used the same likelihood ratios and priors as in Figure 3*a–c*, but with sensory noise $\hat{x}∼\text{N}(x,0.25$) and $\hat{x}∼\text{N}(x,0.5$), respectively, to match the medium and high noise target widths. The model predicted greater separation between the low-prior (teal) and high-prior (orange) decision curves, i.e., greater prior use, in the high-noise condition than in the medium-noise condition, quantified by the high prior – low prior intercept difference (Fig. 4*c*). In other words, the Bayesian ideal observer used the prior more with increasing sensory noise.

Human participants showed the opposite effect: they used their priors less with increasing noise. Psychometric curves across priors moved closer together in the high-noise (σ_*t*_ = 0.5°) condition (Fig. 4*e*) compared with the medium-noise (σ_*t*_ = 0.25°) condition (Fig. 4*d*). The difference in intercepts was significantly greater in the medium-noise condition (mean = 0.17, SE = 0.04) than in the high-noise condition (mean = 0.06, SE = 0.03; *p* = 0.0081 using a paired *t* test; Fig. 4*f*). As for the prior training trials, we confirmed that the results were similar both in and opposite to the direction of the saccade before pooling data for the final results reported here (Extended Data Fig. 3-1*b*,*c*,*e*,*f*). Results using the Criterion, rather than the intercept, as a measure replicated the findings (Extended Data Fig. 4-1). Overall, the results in Experiment 2 suggested that, in this sense, human participants were qualitatively “anti”-Bayesian.

We considered the possibility that participants were not anti-Bayesian but had learned that trials with medium-noise and high-noise targets had a neutral jump probability. In this case, their prior for the hypothesis-testing trials would be 0.5 and the Bayesian prediction is for the orange and teal psychometric curves to collapse together with increasing noise. Note that if the participants only learned the priors according to target type (i.e., low noise targets = color-cued prior, but medium-noise and high-noise targets = 0.5), then there would be no separation between the orange and teal psychometric curves at all. Therefore, participants clearly learned the color-associated priors. Nevertheless, to account for this potential confound, we analyzed results from a control experiment using two rhesus macaques in which the jump probability matched the color-associated prior for all noise levels. A full description of the monkey experiments is provided in Materials and Methods, Rhesus macaque psychophysics.

For the monkey control experiment, all seven trial types (three priors with low sensory noise + two each with medium noise and high noise) were randomly interleaved and had the same relative frequencies (Fig. 4*g*). Consistent with the human results, for both monkeys the intercept differences between the P(J) = 0.8 and 0.2 conditions decreased with increasing sensory noise (Fig. 4*h*,*i*). Repeated-measures ANOVA on intercept differences with noise levels as the main within-subjects factor yielded significant effects (Monkey S: *F*_(2)_ = 51.75, *p* = 4.97 × 10^−15^; Monkey T: *F*_(2)_ = 4.56, *p* = 0.0176). For monkey S (*n* = 40 sessions), *post hoc* comparisons (Tukey’s HSD) showed that intercept differences in the low noise condition (σ_t_ = 0.5°; mean = 0.82, SE = 0.02) were significantly higher than in the medium-noise (σ_t_ = 1.25°; mean = 0.60, SE = 0.05; *p* = 3.49 × 10^−6^) and high-noise (σ_t_ = 2°; mean = 0.40, SE = 0.05; *p* = 0) conditions. Intercept differences in the medium-noise condition were also higher than in the high-noise condition (*p* = 1.51 × 10^−5^). For Monkey T (*n* = 18 sessions), there was a significant difference between the low-noise (σ_t_ = 0.5°; mean = 0.52, SE = 0.06) and high-noise (σ_t_ = 1.75°; mean = 0.36, SE = 0.05; *p* = 0.0190) conditions. Intercept differences in the medium-noise condition (σ_t_ = 1.25°; mean = 0.39, SE = 0.06) fell between the low-noise and high-noise conditions, not significantly different from either. We also analyzed prior use as measured by the difference in response rates at all displacements rather than just the intercepts and found the same result (Extended Data Fig. 4-2). No matter how measured, prior use decreased with increasing sensory noise.

Note that the monkeys were exposed to comparable numbers of trials with valid priors at higher noise levels in this control experiment than in a separate experiment, discussed below, when they were exposed to neutral priors at higher noise levels (Experiment 4). Specifically, Monkey S was exposed to 2.5 times as many valid-prior trials here (11,946 vs 2998 neutral priors) and Monkey T, nearly as many valid-prior trials here (2435 vs 2856 neutral-prior trials). They had ample opportunity to learn the valid priors, but even so, their prior use decreased with increasing visual noise (Fig. 4*h*,*i*). Further, this valid-prior control experiment was conducted after the neutral-prior Experiment 4. If the animals learned the priors in a noise-dependent way, i.e., learned that the medium-noise and high-noise trials had neutral priors in Experiment 4, then they should have learned the new, valid priors in the same noise-dependent way while performing the control experiment. If so, prior use should have differed in the first and second chronological halves of the control experiment, perhaps even converting to Bayesian prior use in the second half. However, we did not find this (Extended Data Fig. 4-3); the prior use always decreased with increasing sensory noise. Overall, the monkey results replicated and extended our human findings to confirm that the anti-Bayesian effect was based on learned, color-associated priors, rather than priors dependent on the target noise level.

Finally, we also fit the Bayesian ideal observer model to the data using a maximum likelihood estimate to evaluate whether, despite the qualitative deviation in behavior from Bayesian predictions, the model could explain the results if allowed to flexibly fit the data with free parameters (Extended Data Fig. 4-4). We found that the Bayesian ideal observer model was unable to converge on parameters that reasonably recapitulated behavior (Extended Data Fig. 4-4*a–c*). Specifically, although the model outputs matched behavior reasonably well in the low-noise (Extended Data Fig. 4-4*a*) and medium-noise (Extended Data Fig. 4-4*b*) conditions, they systematically overestimated prior use in the high-noise (Extended Data Fig. 4-4*c*) condition. The results and their implications are discussed in greater detail, along with the context of the model’s performance for monkey data in Experiment 4, in Discussion.

### Continuous judgments of displacement are Bayesian

**Figure 5.**
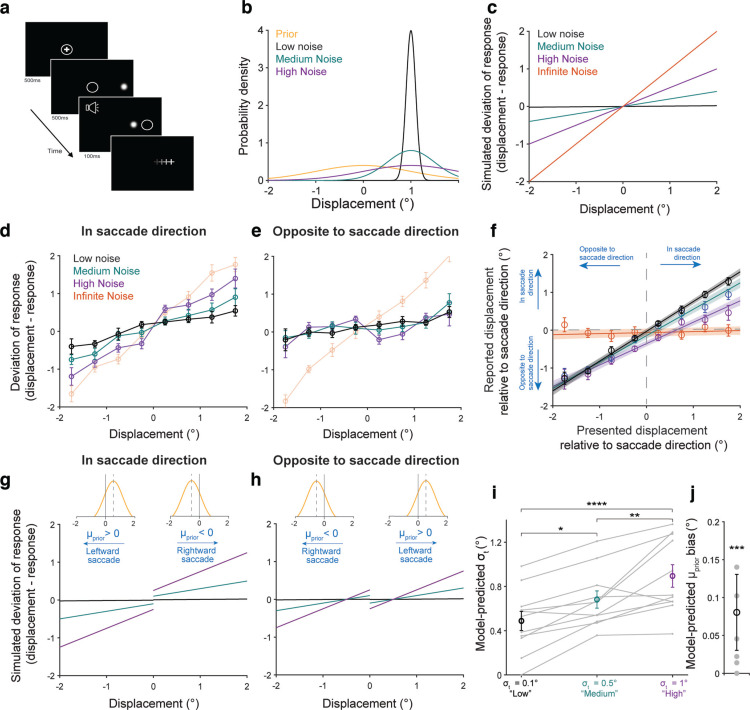
Continuous displacement perception is Bayesian. ***a***, Task schematic. Participants performed the same SSD task as in Experiments 1 and 2 but provided a continuous estimate of where the target landed after the saccade using a mouse cursor (+). ***b***, Distributions used in the experiment. Distributions for the three noise levels are centered on displacement = 1° for illustration. ***c***, Bayesian predictions for the experimental parameters in ***b***. ***d***, ***e***, Results from *n* = 11 participants for displacements in the direction of the saccade (***d***) and opposite to the direction of the saccade (***e***). Bins were averaged across participants and connected with lines. Error bars: SEM. ***f***, Presented versus reported displacements relative to the direction of the saccade (positive = in saccade direction, negative = opposite to saccade direction). Lines were fit to individuals and averaged across participants. Shaded region: SEM. Participants exhibited a response bias opposite to the direction of the saccade. ***g***, ***h***, Bayesian predictions with biased priors (against the direction of the saccade, as observed in ***f*** for displacements in the saccade direction (***g***) and opposite to the saccade direction (***h***). ***i***, ***j***, Model fits for participants’ internal likelihood distribution SDs (***i***) and prior means (***j***). *p < 0.05, **p < 0.01, ****p < 0.0001. Extended Data Figure 5-1 shows the results of incorporating the observed bias into the categorical Bayesian ideal observer model.

Do the above results mean that the perception of visual displacement across saccades is always anti-Bayesian? Or was that outcome due, at least in part, to the categorical (binary) nature of the task? We tested this in Experiment 3 by requiring continuous estimates of displacement across saccades (Niemeier et al., 2003). Human participants performed the same SSD task, but instead of providing a binary report of “jumped” or “did not jump,” they provided a continuous report using a mouse cursor (Fig. 5*a*). The target jumps were horizontal, and the mouse cursor was restricted to that dimension. Formulating the task as a unidimensional, continuous problem allowed us to cast it in a form that has been tested across many sensorimotor domains (Jacobs, 1999; Ernst and Banks, 2002; Kording and Wolpert, 2004; Fetsch et al., 2012; Darlington et al., 2017). If the uncertainty about the stimulus is modeled as the sensory likelihood, then the mean of the posterior (its maximum value and thus, our approximation of the inferred response) would be a reliability-weighted combination of the sensory likelihood and prior distributions:

(26) $$\begin{array}{c} \mu_{posterior}=\frac{\sigma_{likelihood}^{2}\mu_{prior}+\sigma_{prior}^{2}\mu_{likelihood}}{\sigma_{likelihood}^{2}+\sigma_{prior}^{2}} \end{array}.$$

10.1523/ENEURO.0403-22.2023.f5-1 Extended Data Figure 5-1 Incorporating a bias into the categorical Bayesian ideal observer model did not predict the anti-Bayesian results observed in Experiment 2. We modeled an opposite-to-saccade bias for displacements in the categorical Bayesian ideal observer model by shifting the “jump” (red) and “nonjump” (black) distributions from which displacements were drawn. ***a***, For a left saccade, the distributions would shift rightward (indicated by the dashed line) and (***b***) for a right saccade, they would shift leftward. Since we take the absolute value of displacements to compute our logistic psychometric curves, negative (leftward) displacements and the distributions they are drawn from are mirrored about the *y*-axis. ***c***, This resulted in a simulated bias >0 for jumps opposite to the saccade (corresponding to the blue shaded regions in ***a***, ***b***) and (***d***) a bias <0 for jumps in the direction of the saccade (gray regions in ***a***, ***b***). ***e–g***, Simulated psychometric curves with a bias of +0.15° for the (***e***) low-noise, (***f***) medium-noise, and (***g***) high-noise conditions moved further apart with increasing sensory noise. ***h***, Simulated intercept differences in the medium-noise and high-noise conditions (comparable to Fig. 3*e*) quantified the prediction that prior use increased with increasing noise. ***i–l***, Same as in ***e–h*** but with a simulated bias of −15°. Download Figure 5-1, TIF file.

As σ^2^_likelihood_ increases, with the other terms held constant, μ_posterior_ approaches μ_prior_. In other words, for a given prior with fixed uncertainty, the response should get closer to the prior with greater sensory uncertainty.

The prior was a Gaussian statistical distribution with μ_prior_ = 0° and σ_prior_ = 1°. Participants were first trained on the prior for 600 trials using performance-based feedback. They then performed 400 hypothesis testing trials that provided no feedback. There were four sensory uncertainty conditions: low-noise (σ_t_ = 0.1°), medium-noise (σ_t_ = 0.5°), high-noise (σ_t_ = 1°), and an “infinite-noise” condition in which the target did not reappear postsaccadically. Figure 5*b* illustrates the distributions used in the experiment, with the prior centered at 0° and the likelihood (Gaussian blob) distributions centered, for purpose of illustration, on displacement = 1°.

Figure 5*c* shows the predicted deviation in response from the presented displacement (displacement – response) for a Bayesian ideal observer (details in Materials and Methods, Modeling). If the sensory uncertainty is much smaller than that of the prior, as in the lowest noise condition (black line), then the deviation of the posterior (response) from the true displacement should be near 0 for all presented displacements. Conversely, for maximal sensory uncertainty as in the infinite-noise condition (orange line), the response should always be the mean of the prior. Since μ_prior_ = 0°, the deviation for each displacement equals the displacement itself. The medium-noise (teal) and high-noise (purple) conditions are predicted to fall in between the low-noise and infinite-noise conditions, with slopes proportional to noise level. In summary, the slope of the deviation line increases with increasing sensory uncertainty.

As with Experiment 2, we sought to confirm that responses when the target was displaced in the direction of the saccade versus opposite to the saccade were similar before pooling data. In this case, we found that the patten of responses was qualitatively different in the two directions. Figure 5*d*,*e* shows binned responses (*n* = 11 participants), with lines connecting bins, when displacements were in the saccade direction and opposite to the saccade direction, respectively. We made three observations in these results. First, slopes for displacements in the direction of the saccade appeared to increase with increasing sensory noise as predicted in Figure 5*c*, while they collapsed for displacements opposite to the saccade. Second, despite this difference, responses in the infinite-noise condition were similar for both subsets of the data. In the infinite-noise condition, the deviation from the presented displacement closely tracked the unity line, suggesting that participants were reverting to reporting the presaccadic location of the target (i.e., displacement = 0). Finally and critically, although the Bayesian ideal observer model predicted lines with increasing slopes (Fig. 5*c*), the data did not appear to be smoothly linear in either condition. There was a discontinuity in the lines at displacement = 0 that followed opposite trends. This discontinuity is most apparent in the high-noise condition (purple lines).

What is a possible explanation for both the difference in behavior depending on the displacement direction relative to the saccade, and the discontinuity at 0? First, such a discontinuity might arise if participants were using a Bayesian model with bimodal priors (e.g., as in Kording and Wolpert, 2004, their Fig. 3). Second, participants might have developed such bimodal prior representations if they had a natural bias either toward or opposite to the saccade direction. If so, when the data are split by the direction of the displacements relative to the saccade, then the spatiotopic priors (i.e., rightward displacements = positive, leftward = negative) would be bimodally biased away from 0 for both subsets of the data. To investigate further, we re-categorized positive and negative displacements as being in the direction of or opposite to the saccade, respectively. Then, we plotted the reported displacement against the presented displacement (Fig. 5*f*). We found that participants did exhibit a bias opposite to the direction of the saccade (i.e., lines did not pass through 0) that scaled with sensory noise, consistent with the possibility that they were integrating a slightly biased prior with sensory evidence in this task. This suggested that participants’ internal representation of the prior (experimentally centered at 0) was <0 (i.e., left-shifted) for rightward saccades and >0 (i.e., right-shifted) for leftward saccades.

Next, we simulated Bayesian ideal observer predictions for data split by the direction of the displacement relative to the saccade using biased priors (prior mean shift = ±0.5° opposite to the saccade direction, prior SD = 1°). Bayesian predictions for displacements in the direction of the saccade are shown in Figure 5*g* and for displacements opposite to the direction of the saccade in Figure 5*h*. We found that Bayesian predictions using priors biased opposite to the direction of the saccade, as found in Figure 5*f*, qualitatively captured the patterns observed in the data (compare Fig. 5*g* to *d* and *h* to *e*). Specifically, the Bayesian predictions recapitulated the patterns of discontinuity observed in both subsets, as most clearly observed for the high-noise condition (purple lines). Note that since responses in the infinite-noise condition reverted to reporting the presaccadic location, we excluded them from these simulations for comparison.

Finally, we fit individual participants’ responses to a Bayesian ideal observer model by minimizing squared error to infer their used prior mean and sensory likelihood distributions. The prior mean and SDs of the low, medium, and high noise were fit simultaneously. We assumed that the bias opposite to the direction of the saccade was symmetrical on the left and right side of the screens for simplicity. Fit parameters for likelihood SDs increased with increasing noise, with repeated-measures ANOVA yielding a main factor of noise level (*F*_(2)_ = 20.18, *p* = 0.00001; Fig. 5*i*). *Post hoc* comparisons (Tukey’s HSD) showed that the fit SDs in the low noise condition (0.49 ± 0.09) were significantly lower than in the medium-noise (0.68 ± 0.08; *p* = 0.0176) and high-noise (0.89 ± 0.10; *p* = 0.000009) conditions. Similarly, SDs in the medium-noise condition were significantly lower than in the high-noise condition (*p* = 0.0090). Model outputs for the prior revealed a significant bias of 0.08 ± 0.05° opposite to the saccade (*p* = 0.0009, one-sample Wilcoxon signed-rank test; Fig. 5*j*).

Overall, the results of Experiment 3 showed that when participants were required to make continuous estimates of displacement across saccades, their responses matched the predictions of a Bayesian ideal-observer model with biased priors. To test whether the opposite-to-saccade bias for continuous displacements might predict anti-Bayesian performance in the categorical task, we incorporated a bias into the categorical Bayesian ideal observer model (Extended Data Fig. 5-1). Bias was modeled by allowing the displacement distributions from which “jump” and “nonjump” trials were drawn to be shifted away from 0. However, shifting the distributions did not qualitatively change the predictions of the model, suggesting that the continuous bias did not explain the surprising anti-Bayesian performance in Experiment 2.

### Anti-Bayesian categorization is driven by image noise but not motor-driven noise

The above results showed that continuous perception across saccades is Bayesian, but categorical perception is anti-Bayesian. What gives rise to this puzzling dichotomy? Since behavior in other categorical tasks often is Bayesian (Wald, 1945; Ratcliff, 1978; Roitman and Shadlen, 2002; Bitzer et al., 2014; Hanks et al., 2014), our findings are likely more related to the perceptual system we studied than the task structure. In the visual system, object location is signaled via the organization of spatial receptive fields. Receptive fields are continuous from the retina to higher order visual areas (Colby et al., 1988; Engel et al., 1997; Golomb and Kanwisher, 2012a; Arcaro and Livingstone, 2017) and maintain their retinotopic properties even across eye movements (Golomb and Kanwisher, 2012b; Zimmermann et al., 2013) and when remapped (Hall and Colby, 2011; Neupane et al., 2020; Golomb and Mazer, 2021). Moreover, neurons in the frontal eye field use continuous tuning to represent object displacements across saccades (Crapse and Sommer, 2012), the stimulus quantity we probed directly. Thus, the intrinsic organization for processing visual location across saccades is in continuous coordinates. If reports of displacement are required in similarly continuous coordinates, the visual system is perhaps well equipped to use a Bayes optimal strategy. Requiring a categorical report of the continuous system might necessitate an alternative strategy. This explanation has two important implications.

**Figure 6.**
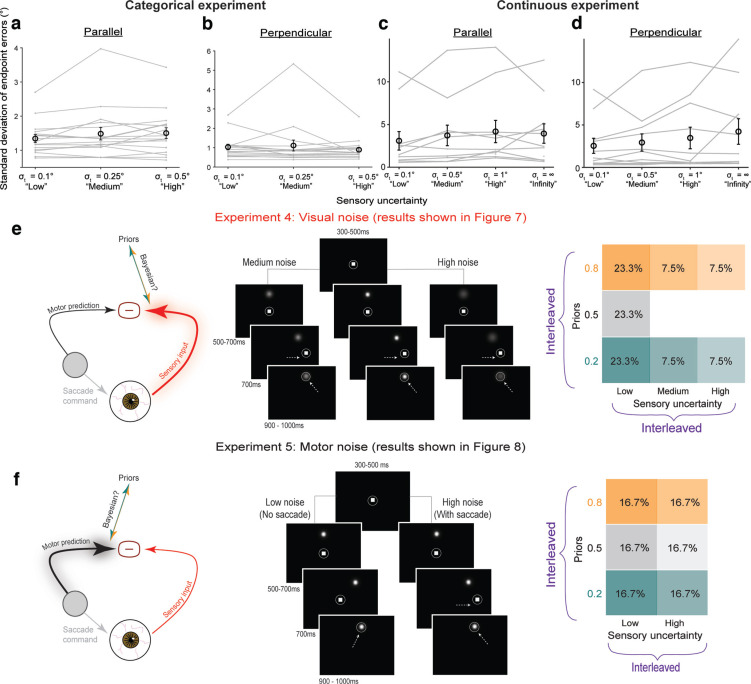
Image noise versus motor-driven noise. ***a–d***, Saccade endpoint error scatter across the three image noise levels in Experiments 2 (Categorical) and 3 (Continuous), as quantified in the directions (***a***, ***c***) perpendicular and (***b***, ***d***) parallel to the saccade. ***e***, ***f***, In monkeys, we separately tested how prior use changes with (***e***) image noise in Experiment 4 and (***f***) motor-driven noise in Experiment 5; for each experiment, the rationale (left), task events and stimulus configurations (middle), and trial breakdown (right) are schematized.

First, it implies that anti-Bayesian prior use was driven primarily by the organization of the visual system. A potential counterargument is that the Gaussian blob in Experiments 2 and three was both the visual object and the saccade target. Blurring it might have added noise to the saccade endpoint relative to the target and consequently the motor prediction (Fig. 1*a*, black arrow), which depends on a copy of the saccade command, in addition to adding noise to the visual input (Fig. 1*a*, red arrow). We did not find evidence of this, however. As a function of Gaussian blur, the SDs of saccadic endpoint errors (endpoint – target location; van Opstal and van Gisbergen, 1989; van Beers, 2007) did not change either parallel or perpendicular to the saccade in either the categorical (Experiment 2; Fig. 6*a*,*b*) or the continuous (Experiment 3; Fig. 6*c*,*d*) experiment for humans (repeated-measures ANOVAs: *F*_(2)_ = 3.1, *p* = 0.0588 in Experiment 2 and *F*_(3)_ = 1.55, *p* = 0.2230 in Experiment 3 for endpoints parallel to the saccade; *F*_(2)_ = 0.97, *p* = 0.3912 in Experiment 2 and *F*_(3)_ = 2.6, *p* = 0.1129 in Experiment 3 for endpoints perpendicular to the saccade). Therefore, uncertainty in the visual input seems to have been the sole factor driving the anti-Bayesian prior use.

**Figure 7.**
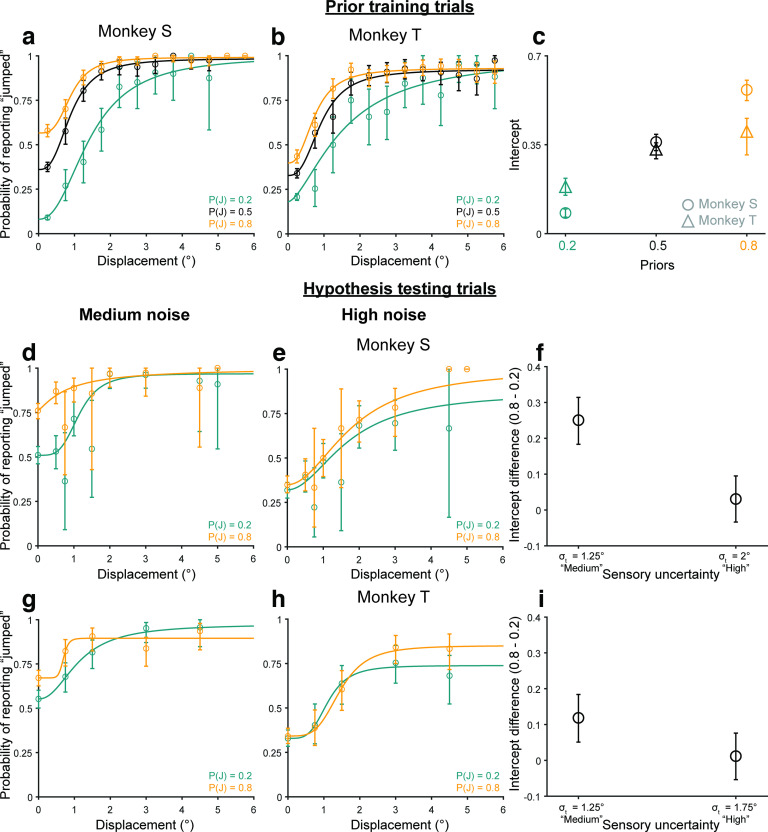
Monkeys were anti-Bayesian for image noise. Monkey S: 10,130 trials from nine sessions. Monkey T: 9958 trials from 18 sessions. 95% confidence intervals bootstrapped from 10,000 samples. ***a–c***, Both animals’ performance in prior learning trials in terms of psychometric curves (***a***: Monkey S; ***b***: Monkey T) and intercept differences (***c***: both monkeys) matched the predictions of the Bayesian ideal observer model in Figure 3*d*,*e*. ***d–i***, Prior use for both monkeys (***d–f***: Monkey S; ***g–i***: Monkey T) was anti-Bayesian. They showed greater prior use in the medium-noise condition (***d***, ***g***) than in the high-noise condition (***e***, ***h***), as quantified by the intercept differences (***f***, ***i***). Extended Data Figures 7-1 and 7-2 show psychometric curves split by the direction of the displacement relative to the saccade direction for Monkeys S and T, respectively. Extended Data Figure 7-3 shows that the results replicate when using Criterion instead of the intercept as a measure of prior use. Extended Data Figures 7-4 and 7-5 show the results of fitting the Bayesian ideal observer model to the data in Experiment 4 for Monkeys S and T, respectively.

10.1523/ENEURO.0403-22.2023.f7-1 Extended Data Figure 7-1 The direction of target displacement relative to the saccade did not influence the results of Experiment 4 (Monkey S). ***a–c***, Data from displacements in the direction of the saccade. ***d–f***, Data from displacements opposite to the direction of the saccade. ***a***, ***d***, Data from prior learning trials. The direction of target displacement relative to the saccade did not matter for Monkey S, as seen by comparing the data shown here with the pooled data of Figure 7*a*,*d–f*. Download Figure 7-1, TIF file.

10.1523/ENEURO.0403-22.2023.f7-2 Extended Data Figure 7-2 Same as Extended Data Figure 7-1, but for Monkey T (compare Fig. 7*b*,*g–i*). Again, results were essentially unchanged; the direction of target displacement did not matter. Download Figure 7-2, TIF file.

10.1523/ENEURO.0403-22.2023.f7-3 Extended Data Figure 7-3 Replication of the Experiment 4 (image noise) results using Criterion instead of intercepts. ***a***, ***c***, For both monkeys, Criterion decreased with the prior magnitude, demonstrating that they learned the priors in training trials (***a***: 0.74 [0.67 0.81], −0.11 [−0.17 −0.05], and −0.44 [−0.51 −0.37] for low prior, baseline, and high prior, respectively, for Monkey S, and ***c***: 0.39 [0.32 0.47], −0.08 [−0.13 −0.02], and −0.25 [−0.32 −0.18] for Monkey T). A lower Criterion value meant that participants were more likely to report “jumped.” Plotting conventions as in Figure 7*c*, except for plotting the results for each monkey separately here. ***b***, ***d***, For both monkeys, Criterion difference between low and high priors in the medium-noise condition (***b***: 0.67 [0.51 0.81] for Monkey S and ***d***: 0.37 [0.21 0.50] for Monkey T) were higher than in the high-noise condition (***b***: 0.08 [−0.05 0.21] for Monkey S and ***d***: 0.16 [0.02 0.30] for Monkey T). In other words, the monkeys used their priors less with greater image noise, the same result as when using intercept differences (compare Fig. 7*f*,*i*). Plotting conventions as in Figure 7*f*,*i*. Download Figure 7-3, TIF file.

10.1523/ENEURO.0403-22.2023.f7-4 Extended Data Figure 7-4 Bayesian Ideal Observer model fits to data in Experiment 4 produced anti-Bayesian best-fit parameters (Monkey S). ***a–c***, The model recapitulated the observed patterns in the binned, empirical data for the low-noise (***a***), medium-noise (***b***), and high-noise (***c***) conditions. Although the best-fit output parameters increased with increasing priors [***d***, −0.3, 0.5, and 0.70 for the *P*(*J*) = 0.2, *P*(*J*) = 0.5, and *P*(*J*) = 0.8 conditions, respectively], the output parameters for the sensory noise level were the opposite of those expected by increasing the target blurriness (***e***, 0.64°, 0.64°, and 0.44° for the low-noise, medium-noise, and high-noise conditions, respectively; highlighted by red dashed box). ***f***, Best-fit parameters for widths of the “jump” (3.27°) and “nonjump” (0.0003°) qualitatively matched the directions of the values used in the experiment. The fit lapse rates increased with sensory noise as expected (for low, medium, and hig, respectively, the fit lapse rates were 0.03, 0.08, and 0.36). Download Figure 7-4, TIF file.

10.1523/ENEURO.0403-22.2023.f7-5 Extended Data Figure 7-5 Same as Extended Data Figure 7-4, but for Monkey T. ***a–c***, Again, the model recapitulated the observed patterns in the binned, empirical data for the low-noise (***a***), medium-noise (***b***), and high-noise (***c***) conditions. Although the best-fit output parameters qualitatively increased with increasing priors [***d***, 0.51, 0.53, and 0.54 for the *P*(*J*) = 0.2, *P*(*J*) = 0.5, and *P*(*J*) = 0.8 conditions, respectively], the output parameters for the sensory noise level were the opposite of those expected by increasing the target blurriness (***e***, 0.61°, 0.60°, and 0.08° for the low-noise, medium-noise, and high-noise conditions, respectively; highlighted by red dashed box). ***f***, Best-fit parameters for widths of the “jump” (8.79°) and “nonjump” (2.99°) qualitatively matched the directions of the values used in the experiment. The fit lapse rates largely increased with sensory noise (for low, medium, and high noise, respectively, the fit lapse rates were 0.09, 0.08, and 0.25 for Monkey T). Download Figure 7-5, TIF file.

Second, the explanation that Bayesian prior use occurs in continuous report tasks for the continuously-organized visual system implies the converse: Bayesian prior use should occur in categorical report tasks for systems having categorical properties. Making a saccade is one example. Each saccade poses an inherent, largely categorical sensory uncertainty in the form of saccadic suppression (Zuber and Stark, 1966; Bridgeman et al., 1975; Diamond et al., 2000; Reppas et al., 2002; Thiele et al., 2002; Bremmer et al., 2009; Wurtz, 2018). Visual processing is suppressed when a saccade is made, and not otherwise. This predicts that prior use would be Bayesian to compensate for saccadic suppression.

These considerations suggest a hypothesis that categorical tasks elicit (1) anti-Bayesian prior use if the sensory uncertainty is continuous (here, because it is represented in the continuously organized visual system), but (2) Bayesian prior use if the sensory uncertainty is categorical (here, because it is because of a saccade being made or not). In Experiments 4 and 5, respectively, we tested these hypotheses. We controlled for motor prediction uncertainty covarying with visual uncertainty by separating the blurred visual stimulus from the saccade target. The experiments used monkeys to permit precise eye position measurements with implanted scleral search coils (Robinson, 1963; Judge et al., 1980).

In Experiment 4 (Fig. 6*e*), we selectively manipulated visual uncertainty by varying only the width of the Gaussian blob (i.e., the image noise), while the saccade target remained constant (Fig. 6*e*, middle panel). The structure of Experiment 4 (Fig. 6*e*, right panel) was nearly identical to Experiment 2 in humans: there were three noise levels (low, medium, and high). Low-noise, prior-training trials comprised 70% of all trials, while medium-noise and high-noise hypothesis-testing trials with neutral jump probability of 0.5 comprised 30% of trials. All trial types were randomly interleaved.

In Experiment 5 (Fig. 6*f*), there were two levels of motor-driven uncertainty. In the “high-uncertainty” condition, monkeys made a saccade to a target (to induce saccadic suppression) and reported whether a probe moved or not. In the “low-uncertainty” condition, they withheld the saccade (no saccadic suppression) while the probe moved (Fig. 6*f*, middle panel). With-saccade and no-saccade trials at three prior levels each were randomly interleaved (Fig. 6*f*, right panel).

Results from Experiment 4 replicated the results from Experiment 2: the behavior was anti-Bayesian (Fig. 7). We first confirmed that varying the width of the Gaussian probe selectively induced visual but not motor uncertainty. As expected from separating the visual probe from the saccade target, there were no significant changes in SDs of saccade endpoint errors across noise levels. The SDs [95% confidence intervals] for Monkey S were 1.01 [0.98, 1.06], 0.97 [0.94, 1.03], and 0.96 [0.93, 1.00] for the low-noise, medium-noise, and high-noise conditions. For Monkey T, they were 0.92 [0.86, 1.07], 0.84 [0.76, 1.00], and 0.93 [0.80, 1.19].

In prior learning trials, both animals learned the priors as expected [ P(J) = 0.2, 0.5, or 0.8], leading to an upward shift in psychometric functions for the high [ P(J) = 0.8] prior and a downward shift for the low [ P(J) = 0.2] prior (Fig. 7*a*,*b*). Quantitatively, intercepts increased with increasing priors: 0.08 [0.07, 0.10], 0.36 [0.33, 0.39], and 0.57 [0.53, 0.60] for Monkey S (Fig. 7*c*, circles) and 0.18 [0.15, 0.22], 0.33 [0.30, 0.36], and 0.40 [0.31, 0.45] for Monkey T (Fig. 7*c*, triangles).

In the hypothesis testing trials, just as found for human participants, prior use decreased with increasing noise (Fig. 7*d–i*). Psychometric functions for the 0.2 and 0.8 prior conditions got closer to each other with increasing noise for both Monkey S (Fig. 7*d*,*e*) and Monkey T (Fig. 7*g*,*h*), in contrast to the greater separation with noise predicted by a Bayesian model (Fig. 4*a*,*b*). Intercept differences between the high-prior and low-prior conditions reflected this collapsing of curves (Fig. 7*f*,*i*). Monkey S had an intercept difference of 0.25 [0.18, 0.32] in the medium-noise condition but only 0.03 [−0.03, 0.09] in the high-noise condition. For Monkey T, it was 0.12 [0.05, 0.19] for medium noise and 0.01 [−0.05, 0.08] for high noise.

We performed the same checks on the results as done for the human experiments, namely comparing the findings across displacement directions (in vs opposite to the direction of the saccade) and prior use measures (Criterion vs intercept). First, we found that accuracy in the neutral baseline condition was slightly lower opposite to the direction of the saccade (0.7455 [0.72 0.77] vs 0.6762 [0.65 0.70], in vs opposite to saccade direction for Monkey S, and 0.7506 [0.73 0.77] vs 0.6944 [0.67 0.72], respectively, for Monkey T). The psychometric curves in both directions, however, behaved similarly across prior and noise conditions (Extended Data Figs. 7-1, 7-2). Therefore, we pooled the data for final analyses. Second, analyses using the Criterion difference as an alternative measure of prior use replicated the anti-Bayesian results (Extended Data Fig. 7-3).

Overall, the results showed that both monkeys used their priors less with increasing image noise. Note that the control experiment presented in Figure 4*g–i* also selectively varied the width of the Gaussian blob but not the saccade target, replicating the finding that prior use with increasing external, image uncertainty was anti-Bayesian regardless of task structure.

Finally, as in Experiment 2, we fit the Bayesian ideal observer model to the data (Extended Data Figs. 7-4, 7-5). In this case, the model recapitulated the monkeys’ behavior well (Extended Data Figs. 7-4*a–c*, 7-5*a–c*). However, it did so by converging on sensory noise parameters that trended in the opposite direction than would be expected from a Bayesian fit. That is, the inferred sensory noise parameters decreased (Extended Data Figs. 7-4*e*, 7-5*e*) with increasing noise in the experiment. Thus, results from fitting the model to data were consistent with our empirical finding that prior use with increasing image noise was anti-Bayesian.

**Figure 8.**
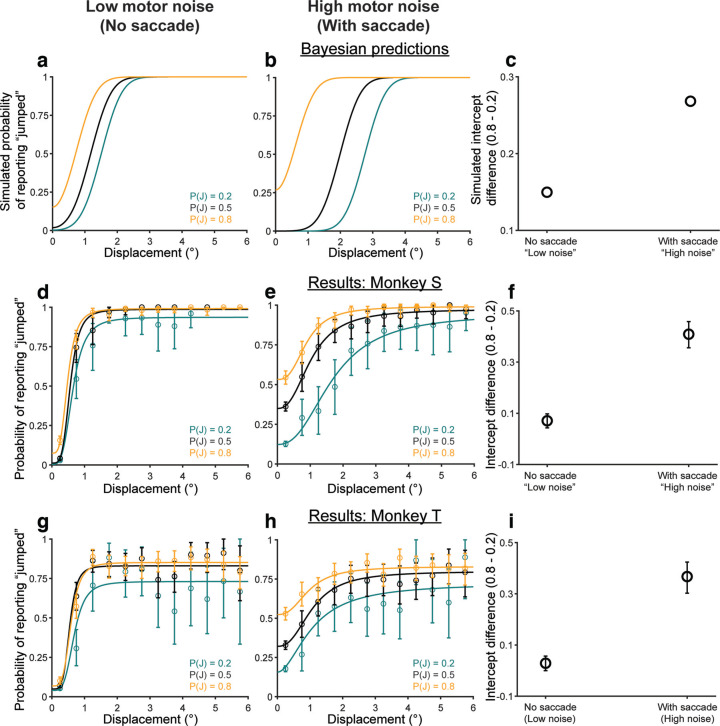
Monkeys were Bayesian for motor-related noise. Top row (***a–c***), Predictions of the Bayesian ideal observer model for two levels of motor-driven noise. Middle row (***d–f***), Results from Monkey S. Lower row (***g–i***), Results from Monkey T. Unlike for image noise (Figs. 4*h*,*i*, 7), the monkeys were decisively Bayesian in their use of priors to compensate for sensory uncertainty introduced by making a saccade. Extended Data Figure 8-1 shows that the results replicate when measuring Criterion. Extended Data Figure 8-2 shows psychometric curves split by the direction of displacement relative to the saccade direction.

Experiment 5 showed, in contrast, that prior use to account for motor-related noise in the categorical task was Bayesian (Fig. 8). Since early visual processing and sensitivity to displacements are suppressed around the time of saccades, we simulated motor-driven noise by increasing the SD of the nonjump likelihood distribution in the with-saccade condition relative to the no-saccade condition (σ_NJ_ = 1° and 0.25°, respectively) while holding the SD of the jump likelihood distribution constant (σ_J_ = 5°). That is, larger displacements are perceived as “nonjumps” in the with-saccade condition to mimic the Saccadic Suppression of Displacement.

10.1523/ENEURO.0403-22.2023.f8-1 Extended Data Figure 8-1 Replication of the Experiment 5 (motor-driven noise) results using Criterion instead of intercepts. For both Monkey S (***a***) and Monkey T (***b***), the Criterion difference in the with-saccade condition (S: 1.21 [1.06 1.36], T: 0.72 [0.58 0.86]) was higher than in the no saccade condition (S: 0.59 [0.38 0.81], T: 0.17 [−0.01 0.33]), indicative of increased prior use with uncertainty and therefore Bayesian behavior. This is the same result found using intercepts (compare Fig. 8*f*,*i*). Download Figure 8-1, TIF file.

10.1523/ENEURO.0403-22.2023.f8-2 Extended Data Figure 8-2 The direction of target displacement relative to the saccade did not influence the results of Experiment 5 (motor-driven noise). As with the categorical image noise experiments (Extended Data Fig. 3-1), the results did not change when data were split by direction of target displacement relative to the saccade. For both Monkeys S (***a–c***) and T (***d–f***), psychometric curves for the different prior conditions were further apart in the with saccade conditions regardless of the direction of the displacement (***b***, ***c***, ***e***, ***f***) than in the no saccade condition (***a***, ***d***). That is, they used their priors more when experiencing motor-driven noise than without motor-driven noise. These are essentially the same results as found when the directions of target displacements were pooled (compare Fig. 8*d*,*e*,*g*,*h*). Download Figure 8-2, TIF file.

The Bayesian model predicts that psychometric functions in the 0.2 and 0.8 prior conditions would separate further (Fig. 8*a*,*b*) and that the difference in intercepts between them would be greater (Fig. 8*c*) with greater motor-driven noise. Results from both monkeys matched these model predictions. Psychometric curves for the different priors showed greater separation when animals made a saccade (Fig. 8*e*: Monkey S; Fig. 8*h*: Monkey T; *n* = 6000 analyzed trials for both animals) than in the condition without a saccade (Fig. 8*d*: Monkey S; Fig. 8*g*: Monkey T). The intercept difference between priors was 0.07 [0.04, 0.10] in the no-saccade condition and 0.41 [0.36, 0.46] when a saccade was made for Monkey S (Fig. 8*f*), and 0.03 [−0.0007, 0.06] and 0.37 [0.30, 0.42], respectively, for Monkey T (Fig. 8*i*). As with Experiment 4, analyses using the Criterion difference as a measure of prior use replicated the results (Extended Data Fig. 8-1). The data were similar for both directions of displacement, i.e., in versus opposite to the direction of the saccade (Extended Data Fig. 8-2).

### A discriminative model provides a candidate explanation for anti-Bayesian categorization

**Figure 9.**
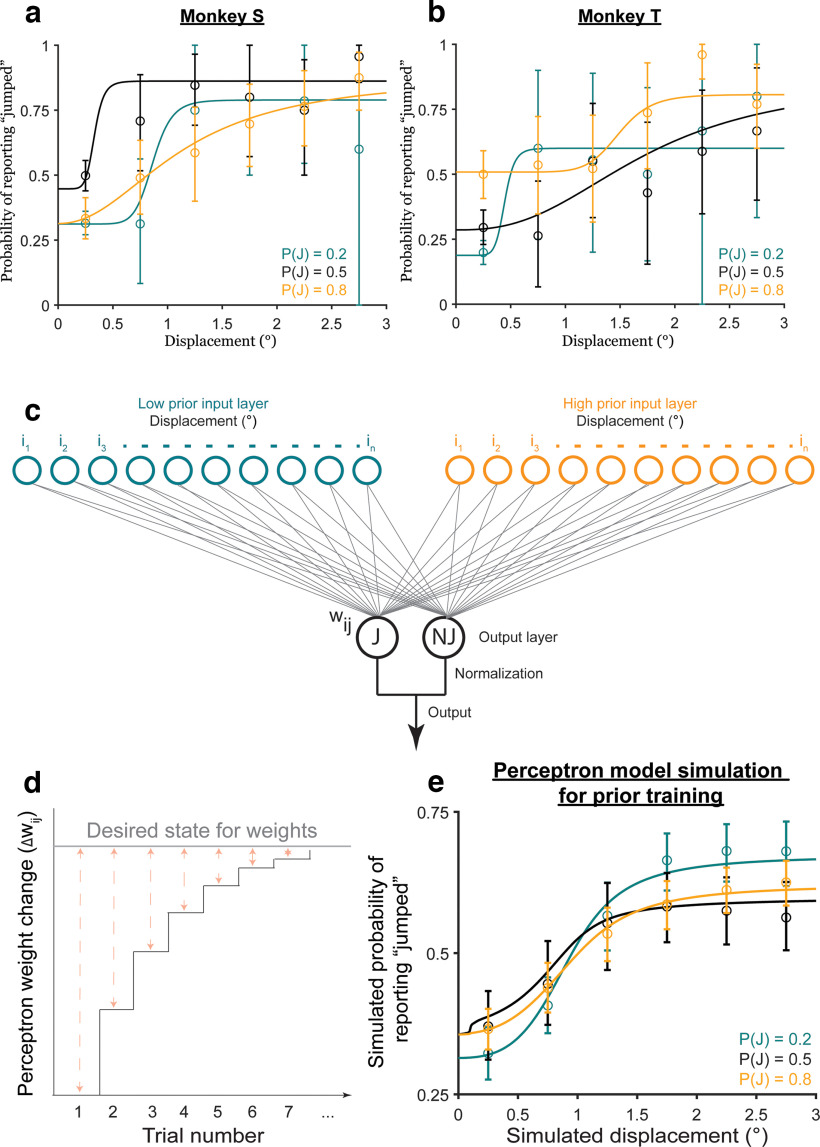
Discriminative (Perceptron) learning model. ***a***, ***b***, Early training results (Monkey S trials 1–500 and Monkey T trials 1–350 for each prior). ***c***, Schematic of the discriminative model. ***d***, Change in weights with each trial. ***e***, Simulations of prior learning under the same conditions as the prior training trials in Experiment 2 on humans. Bins: averaged across 10,000 simulations. Error bars: 95% CI. Psychometric curves are averaged across the simulations (blips at small displacements are an artifact of averaging across different inflection points).

In sum, categorical judgments were Bayesian for motor-driven noise but anti-Bayesian for image noise. Importantly, although we describe behavior that was the opposite of Bayesian ideal observer predictions as being “anti-Bayesian,” we do not intend to imply the existence of mechanisms dedicated to violating Bayesian principles. Instead, the results imply that another process, outside of Bayesian mechanisms altogether, contributes to the perception of stability across saccades. To address this, we separately considered two aspects of the results across humans and monkeys that violated Bayesian predictions. First, prior use decreased with increasing image noise. Second, for human participants, the low prior (teal) curve rose above the baseline (black) curve in Figure 3*f*, violating the Bayesian prediction of parallel prior psychometric curves (Fig. 3*d*). Although prior curves were parallel for monkeys (Figs. 7*a*,*b*, 8*e*,*h*), this was after extensive training. Humans performed only single sessions. The early prior-training data from both monkeys were consistent with the human data (Fig. 9*a*,*b*, teal curves rose above black curves). Therefore, an alternative model would have to explain the disproportionately high “jump” response rate for low priors early in training in addition to the decrease in prior use with increasing visual uncertainty.

An alternative framework to Bayesian models is discriminative models (Rumelhart et al., 1986; Hinton, 1992; Ng and Jordan, 2002; Murphy, 2013), which directly learn to classify stimuli. For our categorical task, a discriminative model would seek to classify continuous displacements into two categories, “jump” and “no jump.” We set up a simple two-layer neural network to classify displacements (Fig. 9*c*) and simulated its performance under experimental conditions.

#### Model structure

The input layer consisted of units representing continuous displacements and the output layer had two units: “jump (J)” and “no jump (NJ).” For ease of computation, continuous input displacements were discretized into bins of 0.1°. Displacements ranged from 0° to 7.5°. That is, there were 75 input units for each network. Sensory noise was simulated as a Gaussian distribution of input unit activation, truncated at the two ends of the input range (0 and 7.5), such that the total activation of input units was always 1. On each trial, the distribution was centered on the true displacement for the trial and the width of activation was determined by the sensory noise level. Each input unit was connected to both output units. The activation of each output unit was the weighted sum of inputs, i.e.,

(27) $$\begin{array}{c} a_{j}=\overset{n}{\underset{i=1}{\sum }}a_{i}w_{ij} \end{array},$$

where $a_{j}$ is the activation of the output unit, j; $a_{i}$ is the activation of the output unit, i; and $w_{ij}$ is the weight of the connection between input unit, i, and output unit, j. The final output on each trial was the normalized activation of the “jump” and “no jump” output units such that the output for each unit was bounded between 0 and 1:

(28) $$\begin{array}{c} o_{jump}=\frac{a_{jump}}{a_{jump}+a_{nojump}} \end{array}$$

and

(29) $$\begin{array}{c} o_{nojump}=\frac{a_{nojump}}{a_{jump}+a_{nojump}} \end{array},$$

where $o_{jump}$ is the final output of the “jump” unit, $o_{nojump}$ is the final output of “no jump” unit, and $a_{jump}$ and $a_{nojump}$ are activations of the “jump” and “no jump” output units, respectively.

The “knowledge” of the two categories is stored in the weights between the input and output units, and the learned, category boundary takes the form of a psychometric function reporting the probability that the target “jumped” given an input displacement. In other words, the shape of the psychometric function is determined by the activation of the inputs and the weights between inputs and outputs. Since psychometric curves have different shapes across priors at the same point in training (e.g., Fig. 3*f*), we assumed that the connections between inputs and outputs (and their corresponding weights) are prior-dependent. This is equivalent to the idea that distributions are learned separately across cue color contexts. We simulated the prior-dependence of the input-output relationship by simply setting up separate sets of inputs for each prior (low-prior and high-prior inputs illustrated in Fig. 9*c*; simulations also included a baseline *P*(*J*) = 0.5 condition).

We next considered how the weights between inputs and outputs might be updated. One possibility was that they are updated by a simple Hebbian-like associative learning rule (Hebb, 1949) where weights between two units are updated in a manner proportional to their activation. We chose a slight variation of this rule based on work by Gluck and Bower (1988), who showed that an error*-*based learning rule, rather than a purely associative learning rule, leads to a disproportionate overweighting of infrequent events early in training.

The learning rule is given by:

(30) $$\begin{array}{c} \Delta w_{ij}=\beta a_{i}\left(d-o_{j}\right) \end{array},$$

where $\Delta w_{ij}$ is the change in weights between input unit, i, and output unit, j; β is the learning rate, $a_{i}$ is the activation of the input unit, i; $o_{j}$ is the final output of unit j, and d is the desired state of output unit, j. The term $\left(d-o_{j}\right)$ is therefore the error between the current output of the model and the desired state determined by feedback on each trial. In summary, the change in weights or learning on each trial is proportional to the activity of the input and the error of the model on that trial. This is equivalent to the Perceptron learning rule (Rosenblatt, 1957; Minsky and Papert, 1969). For infrequent events such as large displacements in the low prior condition, the weight changes between the event and the output early in training are relatively large (Fig. 9*d*, left side). Taking a snapshot of performance at this stage would thus result in an apparent overweighting of their contribution to the output as seen early in training in Figures 3*f*, 9*a*, and 9*b*. This rule, however, predicts that once weights asymptote toward the desired state late in training (Fig. 9*d*, right side), events should contribute to the model’s performance in a manner proportional to their relative frequencies, and psychometric curves should become parallel to one another as seen in Figures 7*a*,*b*, 8*e*,*h*.

#### Simulations

We evaluated the model’s ability to explain the results by simulating its performance under experimental conditions. 95% confidence intervals for each simulated estimate were obtained by running 10,000 simulations and identifying the middle 95% of each estimate, i.e., 2.5 percentile – 97.5 percentile. We simulated early prior training by following the same experimental structure as for the human experiments: a baseline block at P(J) = 0.5 followed by two 600-trial blocks at P(J) = 0.8 and P(J) = 0.2, respectively. Of those trials, 70% were prior training trials at the lowest noise level, σ_target_ = 0.1°. The remaining 30% were medium-noise (σ_target_ = 1°) and high-noise (σ_target_ = 2°) testing trials with a neutral movement statistic of 0.5 but simulated using the same inputs as the prior condition. Displacements were drawn from overlapping Gaussian distributions as in the experiments. Jumps were drawn from a distribution with μ_jump_ = 0°, σ_jump_ = 2.5°, and nonjumps were drawn from a distribution with μ_non jump_ = 0°, σ _non jump_ = 0.5°. On trials where the target jumped, the desired state was set to 1 for the “jump” output unit and 0 for the “no jump” output unit. On trials where the target did not jump, it was set to 0 for the “jump” unit and 1 for the “no jump” unit. The learning rate was set at 0.5. As expected, the outputs recapitulated the disproportionately high response rate for large displacements in the low prior condition (Fig. 9*e*, teal curve). However, it also downweighted the infrequent small displacements in the high prior condition (Fig. 9*e*, orange curve). To account for this, we considered that reports in the categorical task may result from a combination of a discriminative and a Bayesian model. The Bayesian prior use for high saccade-driven uncertainty raises high prior intercepts (Fig. 8*e*,*h*) and thus could compensate for the downweighting by the discriminative model.

**Figure 10.**
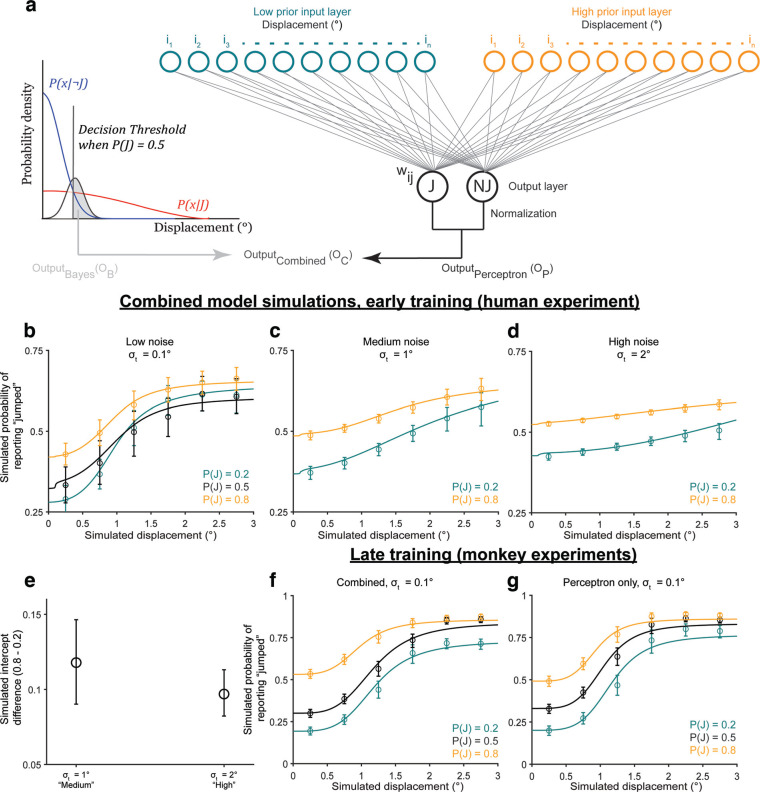
Combined Bayesian and discriminative model. ***a***, Schematic of the Bayesian model output (from Fig. 3*a*) on the left being combined with the output from the discriminative (Perceptron) model. ***b–d***, Psychometric curves simulated under the same experimental conditions as the human experiment for (***b***) low-noise, (***c***) medium-noise, and (***d***) high-noise levels. Bins: averaged across 10,000 simulations. Error bars: 95% CI. Psychometric curves are averaged across the simulations (blips at small displacements are an artifact of averaging across different inflection points). ***e***, Intercept differences across the medium-noise and high-noise conditions. ***f***, ***g***, Late training data for (***f***) the combined model and (***g***) the discriminative model alone.

Therefore, we next simulated a combined model whose final output was a weighted combination of outputs from the discriminative model, which incorporated visual noise, and a Bayesian ideal observer model which incorporated motor-driven noise (Fig. 10*a*). Motor noise was simulated only in the Bayesian model by setting the width of the nonjump distribution, σ_non jump_ = 1.5°, i.e., triple the width of the simulated experimental distribution to mimic saccadic suppression. Bayesian and discriminative model outputs were combined linearly, such that:

(31) $$\begin{array}{c} O_{C}=w_{B}O_{B}+w_{P}O_{P} \end{array},$$

where $O_{C}$ is the output of the combined model, $O_{B}$ is the output of the Bayesian model, $O_{P}$ is the output of the discriminative model, and $w_{B}$ and $w_{P}$ are the weights assigned to the Bayesian and discriminative model, respectively. Further, the weights of the two component models added up to 1:

(32) $$\begin{array}{c} w_{b}+w_{P}=1 \end{array}.$$

We combined the outputs of the Bayesian and discriminative models at relative weights of 0.1 and 0.9, respectively, to generate the data in Figure 10*b*, where the pattern of the curves matched data from human participants well.

Next, adding medium and high noise to the visual input of the discriminative model (but holding visual noise constant in the Bayesian model) caused psychometric curves to move closer to each other with increasing noise (Fig. 10*c*,*d*), as quantified by the downward trend in high-low prior intercept differences (Fig. 10*e*). Finally, we tested the prediction that prior curves become parallel once weights approach a relatively stable desired state for all input units (Fig. 9*d*, right side) by letting the model run for 5000 trials. Data from trials 3000–5000 for the combined (discriminative + Bayesian) model (Fig. 10*f*) and for the discriminative model alone (Fig. 10*g*) support this prediction.

In summary, the combined model recapitulated both the surprising trade-off between priors and noise and the long-term learning effects that were unexplained by a Bayesian ideal observer model alone. This demonstrated that a discriminative learning rule provides a feasible explanation for the anti-Bayesian results, and that the categorization of object displacement across saccades is governed by both Bayesian and discriminative processes.

## Discussion

We found that human participants were Bayesian for continuous reports of object displacement across saccades but anti-Bayesian for categorical reports. Further investigation in monkeys showed that the anti-Bayesian effect was primarily because of external, image noise rather than motor-driven noise. Fitting the Bayesian ideal observer model to the data either failed to recapitulate behavior in Experiment 2 (humans) or failed to yield reasonable parameters in Experiment 4 (monkeys). Instead, the use of a Perceptron-like, discriminative learning rule provided a plausible and parsimonious candidate explanation for anti-Bayesian performance in the categorization task, and a model combining Bayesian and discriminative processes best recapitulated overall performance in the task.

Interestingly, the Bayesian ideal observer model failed in different ways for humans and monkeys in Experiments 2 and 4, respectively. This difference was related to the early-training effect highlighted in Figure 9. For humans, who were at an early stage in prior training, the slope of the low prior curve in the lowest noise condition was disproportionately high. The model likely calibrated its parameters (e.g., via the widths of the “jump” and “nonjump” likelihood distributions) to meet the demands of this increased slope in the lowest sensory noise condition (Extended Data Fig. 4-4*a*) from which the majority of trials were drawn. To then try and match the decreased slope in the highest noise condition (Extended Data Fig. 4-4*c*), it estimated higher noise levels as expected (Extended Data Fig. 4-4*e*). However, given the constraints of a Bayesian ideal observer model structure, estimating higher noise levels requires that the prior curves grow further apart (as seen in the simulations in Fig. 4*a*,*b*), thus causing it to overestimate their separation in the high-noise condition. For monkeys who were at a later prior-training stage, on the other hand, behavior in the low noise condition appeared Bayesian-like. It was only by evaluating prior use against increased noise that we could distinguish Bayesian from Discriminative behavior at this stage. Therefore, the model achieved a qualitatively better match to these data, but with opposite noise parameters.

Limitations of the experimental design and modeling choices should be considered while interpreting the results. First, it is possible that the anti-Bayesian result is a consequence of how we conceptualized parameters (e.g., the prior, or sensory noise) in the categorical Bayesian ideal observer model. For example, for continuous tasks, it has been shown that if the sensory likelihood is asymmetric in a way that can result from assumptions of efficient sensory encoding, then the outcome of a Bayesian decoding process can be seemingly anti-Bayesian (Wei and Stocker, 2015). Of course, alternative parameters might predict the surprising results. However, we chose simulation parameters to closely map onto experimental parameters and mimic empirical phenomena such as saccadic suppression. As a result, the model captures both the Bayesian trade-off with motor-driven noise and the anti-Bayesian trade-off with visual noise. To our best estimate, there was no simple set of alternative parameters that did so parsimoniously.

Second, we limited the simulation of motor-induced noise in Experiment 5 to just one phenomenon, i.e., saccadic suppression. We did this by increasing the width of the nonjump likelihood, P(x|¬ J). We focused on saccadic suppression since it is largely a categorical form of uncertainty that is present when a saccade is made, and not otherwise. It was thus sufficient for testing our hypothesis. However, there are other ways in which saccades influence vision both at the level of behavior and neurons. Such effects include compression of space toward the saccade target (Honda, 1993; Awater and Lappe, 2006; Hamker et al., 2011; Pola, 2011) or the shifting or smearing of visual receptive fields around the time of saccades (Neupane et al., 2020; Golomb and Mazer, 2021). Indeed, the magnitude of saccadic suppression may vary with saccade amplitude (Stevenson et al., 1986) and the direction in which the probe moves relative to the saccade vector (Niemeier et al., 2003; Crapse and Sommer, 2012). Our results do not preclude the inclusion of additional, fine-grained motor-induced phenomena into the normative model, and the resulting predictions would be testable. Another consideration for Experiment 5 is that in the no-saccade condition of the task, animals fixated a central square for the duration of a trial. We did not prevent the animal from making fixational eye movements such as microsaccades (Martinez-Conde et al., 2004), and saccadic suppression may occur around the time of microsaccades (Bair and O’Keefe, 1998; Hafed and Krauzlis, 2010; Martinez-Conde et al., 2013; Hafed et al., 2015). Although we did not control for microsaccades, the stimulus displacement was not timed to microsaccade onset in no-saccade trials as it was to saccade onset in with-saccade trials. On average, therefore, the influence of (micro)saccadic suppression should be quite low in the no-saccade condition.

Finally, we limited the scope of the discriminative and combined models to provide a candidate alternative to the categorical Bayesian model with minimal additional assumptions. This leaves some unexplained patterns in the data, e.g., overall intercepts across priors decrease with increasing noise for humans (Fig. 4*d*,*e*) and monkeys (Fig. 7*d*,*e*,*g*,*h*) but not for the model (Fig. 10*b–d*). Similarly, for our experimental-like parameters, the model does not capture the complete collapse of prior curves with increasing noise. The model may be extended, however, to include additional components such as Bayesian integration in the continuous input layer (from Experiment 3) to better explain the data. Assumptions about how the components combine may additionally be testable too.

The overall pattern of results in our study poses a fundamental question: what determines the use of Bayesian versus discriminative models for perception? Despite recent efforts to acknowledge the contribution of both Bayesian and non-Bayesian models to perception (Laquitaine and Gardner, 2018; Rahnev and Denison, 2018; Gardner, 2019; Sohn and Jazayeri, 2021; DiCarlo et al., 2021), the field lacks a synthesized, theoretical account of when Bayesian models are used and when they are not. As we noted while motivating Experiments 4 and 5, our results suggest a link between the inherent neural organization of sensorimotor systems and Bayesian behavior. Further clarification of this link would allow our understanding of each to constrain and advance our understanding of the other.

Although our study was specific to visual-oculomotor behavior, we expect that the conclusions extend to other sensorimotor systems. Accounting for self-movement is an issue for almost all sensory modalities, and the integration of movement and sensory signals for active perception has been observed widely in the brain (Crapse and Sommer, 2008; Niell and Stryker, 2010; Keller et al., 2012; Schneider and Mooney, 2018). Understanding the relative contributions of Bayesian and discriminative computations to active vision may guide future studies on how expectations, self-movement, and external sensory information combine for more general forms of active perception.

## Synthesis

Reviewing Editor: Nicholas J. Priebe, University of Texas at Austin Decisions are customarily a result of the Reviewing Editor and the peer reviewers coming together and discussing their recommendations until a consensus is reached. When revisions are invited, a fact-based synthesis statement explaining their decision and outlining what is needed to prepare a revision will be listed below. The following reviewer(s) agreed to reveal their identity: Jorge Otero-Millan. Note: If this manuscript was transferred from JNeurosci and a decision was made to accept the manuscript without peer review, a brief statement to this effect will instead be what is listed below.

Synthesis

Two reviewers and the reviewing editor evaluated the resubmitted manuscript “Bayesian and Discriminative Models for Active Visual Perception across Saccades.” This revision addresses many of the concerns raised previously. Both reviewers agree that the authors have demonstrated that the intercept in the high noise condition is not what would be expected from a Bayesian observer model, and as such this work represents an interesting paradigm to examine deviations from Bayesian processes. Both reviewers also noted that the data are noisy, which led the reviewers to express caution about making strong conclusions from this dataset. There remain, however, outstanding issues that need to be addressed. In particular, it was noted that the assumptions of the models do not appear to match the data, as shown by the biases uncovered in the analysis shown in figure 5f. Further, there was concern about the terminology employed in the manuscript. These issues are detailed below, which you should find useful in revising your manuscript.

Major issues:

1) Is the current Bayesian framework used by the authors appropriate for the data?

A major concern from the reviewers is that there are deviations from a Bayesian model that are not accounted for by the current model. In particular, the reviewers noted the bias shown in figure 5f that depends on the direction of the saccade. This bias should have warranted a re-examination of the model. Could the interpretation of the surprising results shown earlier in the manuscript be a result of model assumptions that are not true? As it stands, the model assumes a prior that peaks at 0 and a likelihood that centers around the true jump, but the data demonstrate that these assumptions cannot both be true. This is a major issues that makes the interpretation of the results difficult.

2) “Anti-Bayesian” terminology

Both reviewers suggested that this terminology be changed. The manuscript currently refers to behavior as “anti-Bayesian.” This is synonymous for saying “behavior is not Bayesian”. In the response to the reviews, the authors wrote: “we are not conjecturing anti-Bayesian mechanisms, we argue that these results imply some other process, outside of Bayesian mechanisms altogether, that contribute to the perception of stability across saccades.” If the interpretation is that there are some additional mechanisms at work whose influence may modulate/over-shadow the Bayesian behavior in the observed way, then that should be clearly expressed to avoid any potential confusion.

One suggestion is simply to state that the results deviate from an ideal Bayesian observer model. This fact itself, without a proper hypothesis test, does not imply that behavior is non-Bayesian.

The term “anti-Bayesian” has been used differently used in the literature. It was consistently used to describe perceptual behavior where biases are pointing away rather than toward the prior (see e.g. Peters et al. 2015, Wei and Stocker 2015, Stengard and van den Berg 2019). The authors are free to use this term as they wish as long as it is clearly defined (which it is). To avoid confusion and to keep the literature concise, the reviewers recommend a change since it is not clear that the benefits of this terminology outweigh the drawbacks.

The Size-Weight Illusion is not anti-Bayesian after all: a unifying Bayesian account, Megan A.K. Peters,Wei Ji Ma, and Ladan Shams

Imperfect Bayesian inference in visual perception, Elina Stengård, Ronald van den Berg

A Bayesian observer model constrained by efficient coding can explain ‘anti-Bayesian’ percepts, Xue-Xin Wei & Alan A Stocker
