# Supplementary figures and images for: Bayesian and Discriminative Models for Active Visual Perception across Saccades
Source: eNeuro. 2023 Jul 20;10(7):ENEURO.0403-22.2023. doi: 10.1523/ENEURO.0403-22.2023 (PMC10368208; doi:10.1523/ENEURO.0403-22.2023)

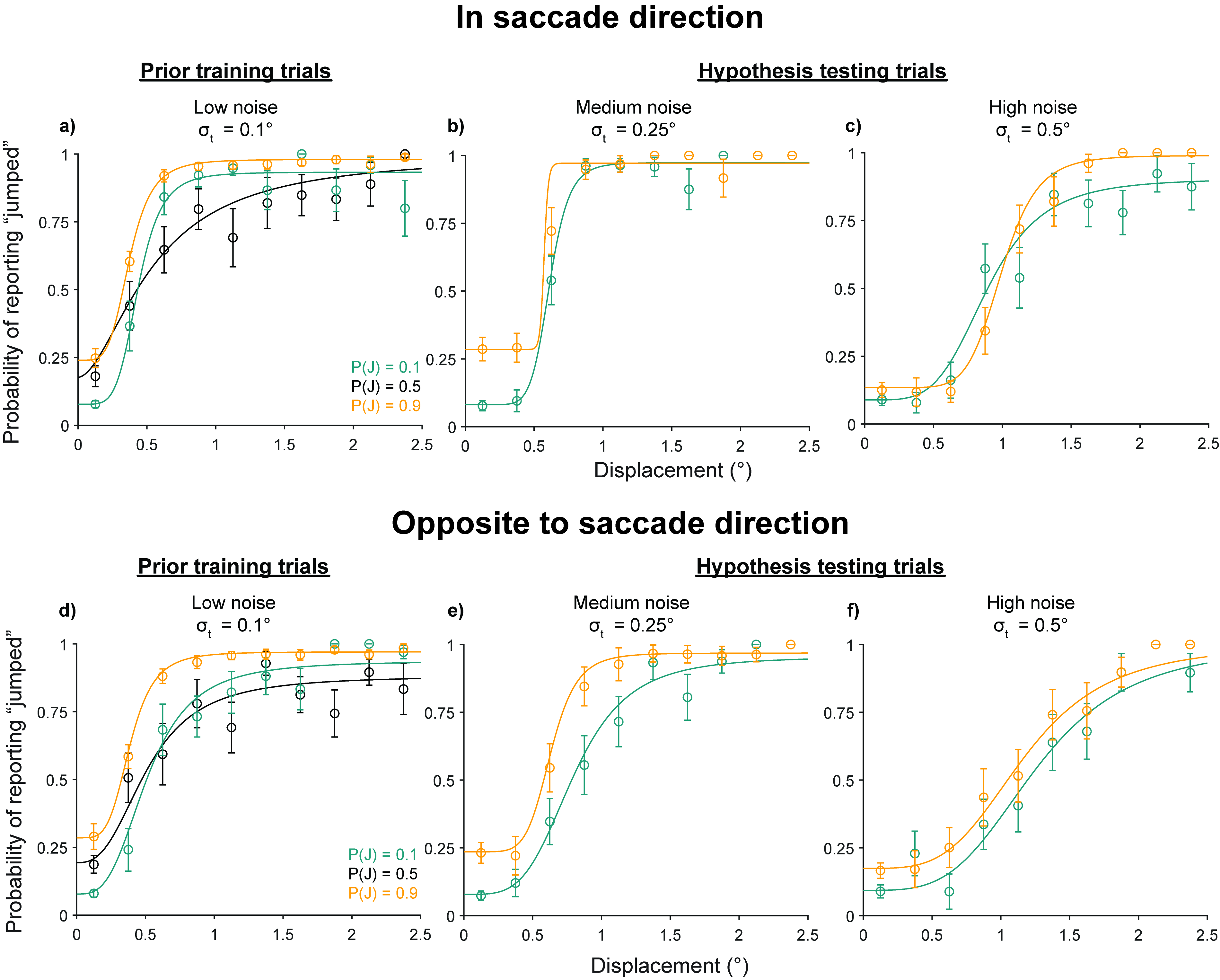

Supplement: Extended Data Figure 3-1 — The direction of target displacement relative to the saccade did not influence the results of Experiment 2. Top, Data from displacements in the direction of the saccade. Bottom, Data from displacements opposite to the direction of the saccade. a, d, Data from prior learning trials. Participants demonstrated that they learned the prior regardless of the direction of the displacement (compare to pooled data in Fig. 3f). b, e, Data from hypothesis-testing, medium-noise trials. c, f, Data from hypothesis-testing, high-noise trials. As with the pooled data (Fig. 4d,e), prior use decreased with increasing noise for both displacement directions. Plotting conventions are the same as in Figures 3 and 4. Overall, the direction of target displacement relative to the saccade did not matter, as seen by comparing the data shown here with the pooled data of Figures 3 and 4. Download Figure 3-1, TIF file. [file enu-eN-NWR-0403-22-s02.tif]

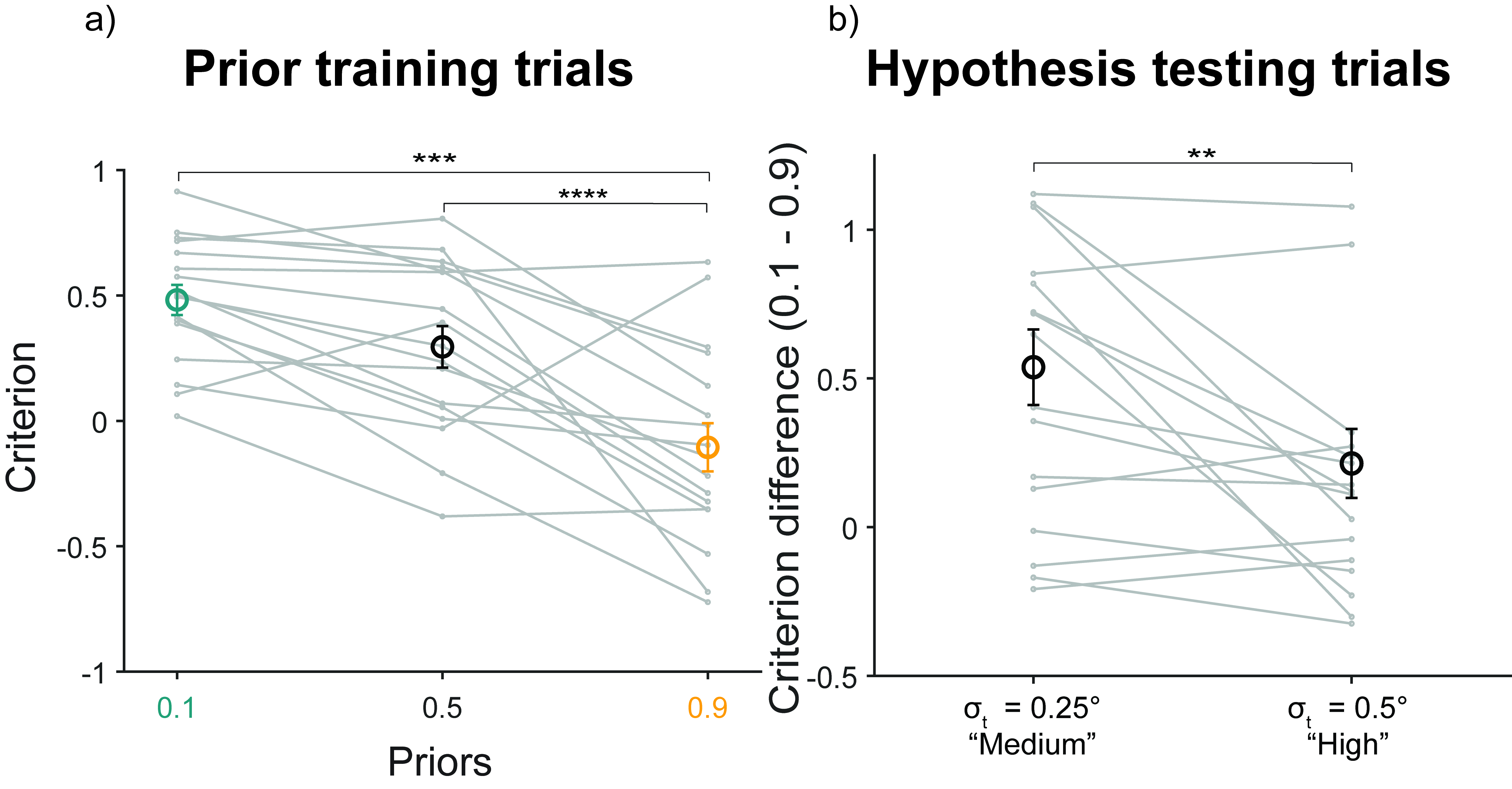

Supplement: Extended Data Figure 4-1 — Replication of the Experiment 2 results using Criterion instead of intercepts. a, Criterion decreased with the prior magnitude, demonstrating that the human participants learned the priors in training trials. Note that a lower Criterion value meant that participants were more likely to report “jumped.” Plotting conventions as in Figure 3g. F(2) = 22.20, p = 8.97 × 10^−7^ on a repeated-measures ANOVA. Post hoc comparisons using a Tukey’s HSD test showed that high prior Criterion values (−0.11 ± 0.10) was significantly lower than baseline (0.30 ± 0.08; p = 0.00028) and low prior (0.48 ± 0.06; p = 7.16 × 10^−7^) values. Low prior and baseline values were not significantly different from each other (p = 0.11137). b, The difference in Criterion values between low and high priors was higher in the medium-noise condition (0.54 ± 0.13) than in the high-noise condition (0.21 ± 0.12; p = 0.0055 on a paired t test). In other words, prior use as measured by Criterion differences decreased as sensory uncertainty increased, the same result as when using intercept differences (compare Fig. 4e). Plotting conventions as in Figure 4e. Download Figure 4-1, TIF file. [file enu-eN-NWR-0403-22-s03.tif]

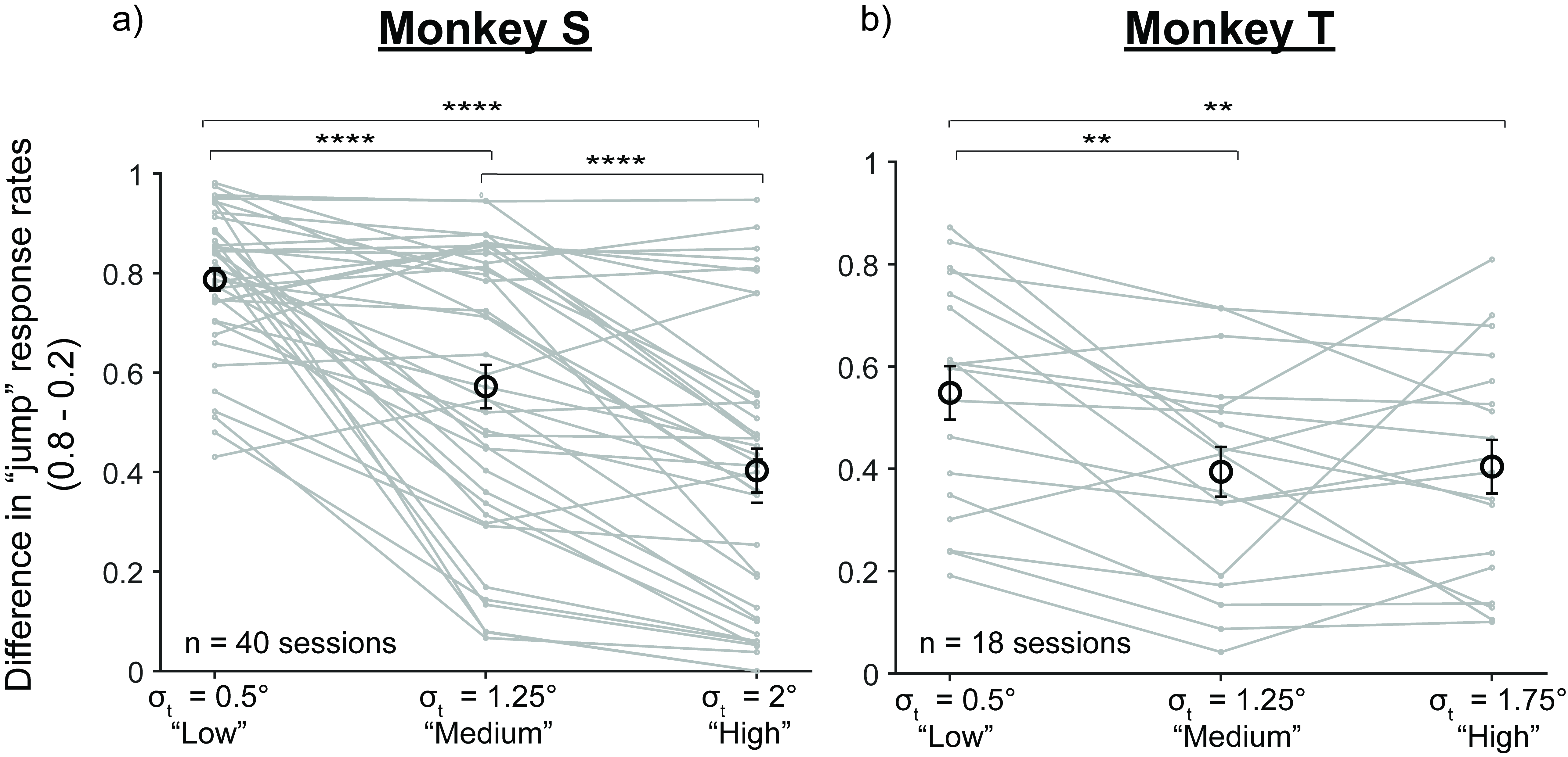

Supplement: Extended Data Figure 4-2 — Replication of the control experiment results using response rates for all displacements instead of intercepts. For both monkeys, S (a) and T (b), the difference in response rates decreased as sensory uncertainty increased (p = 1.57 × 10^−10^ on a Friedman test for Monkey S, and p = 0.0014 on a repeated-measures ANOVA for Monkey T). Post hoc comparisons using pairwise signed-rank exact tests showed that prior use in the high-noise condition (0.40 ± 0.04) was significantly lower than in the low (0.79 ± 0.02; p = 6 × 10^−11^) and medium-noise (0.57 ± 0.04; p = 2.28 × 10^−7^) conditions, and prior use was significantly different in the low-noise and medium-noise conditions (p = 8.85 × 10^−6^) for Monkey S. For Monkey T, post hoc comparisons using Tukey’s HSD tests showed that prior use in the low-noise (0.55 ± 0.05) condition was significantly higher than in the medium-noise (0.39 ± 0.05; p = 0.0030) and high-noise (0.40 ± 0.05; p = 0.0056) conditions. In summary, they used their priors less with greater image noise, the same result as when using intercept differences (compare Fig. 4h,i). Plotting conventions as in Figure 4h,i. Download Figure 4-2, TIF file. [file enu-eN-NWR-0403-22-s04.tif]

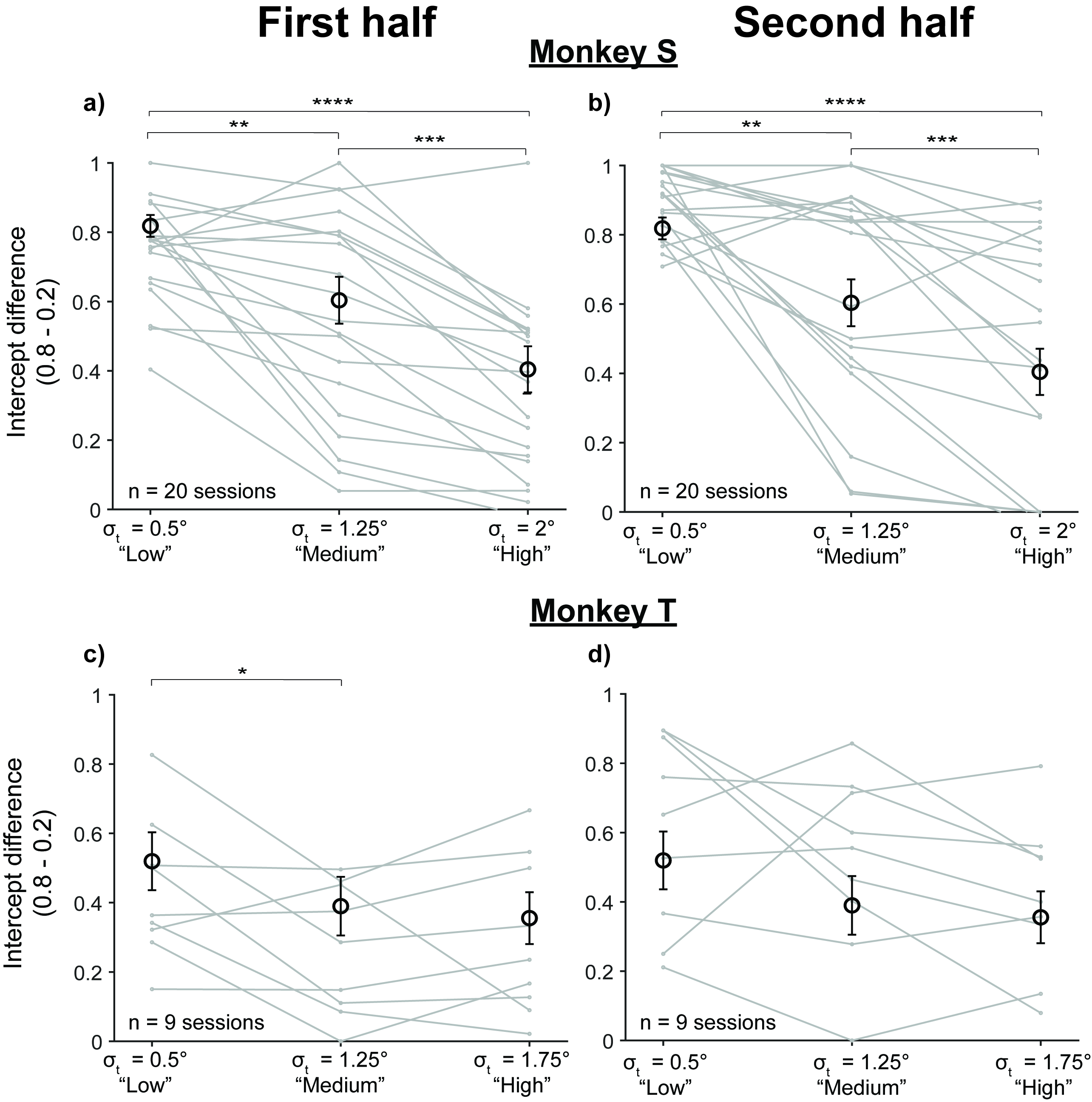

Supplement: Extended Data Figure 4-3 — Prior use decreased with increasing sensory noise within each chronological half of the control experiment. a, b, Results for Monkey S (a, p = 2.28 × 10^−9^ on a repeated-measures ANOVA in the first half; b, p = 1.43 × 10^−6^ on a Friedman test in the second half). c, d, Results for Monkey T [c, p = 0.033 in the first half; d, p = 0.18 in the second half (i.e., decreasing but not significant) on repeated-measures ANOVAs]. For Monkey S in the first half, post hoc Tukey’s HSD comparisons for Monkey S showed that the intercept difference in the low-noise condition (0.75 ± 0.03) was significantly higher than in the medium-noise (0.56 ± 0.07; p = 0.0019) and high-noise (0.35 ± 0.06; p = 2.28 × 10^−5^) conditions, and in the medium-noise condition was significantly higher than in the high-noise condition (p = 0.00025). In the second half (paired signed-rank exact tests), the intercept difference in the low-noise condition (0.89 ± 0.02) was significantly higher than in the medium-noise (0.64 ± 0.07; p = 0.0031) and high-noise (0.46 ± 0.08; p = 9.57 × 10^−5^) conditions, and in the medium-noise condition was significantly higher than in the high-noise condition (p = 0.0014). For Monkey T in the first half, the intercept difference in the low-noise condition (0.43 ± 0.07) was significantly higher than in the medium-noise (0.27 ± 0.06; p = 0.04) condition, but not between the other conditions. Download Figure 4-3, TIF file. [file enu-eN-NWR-0403-22-s05.tif]

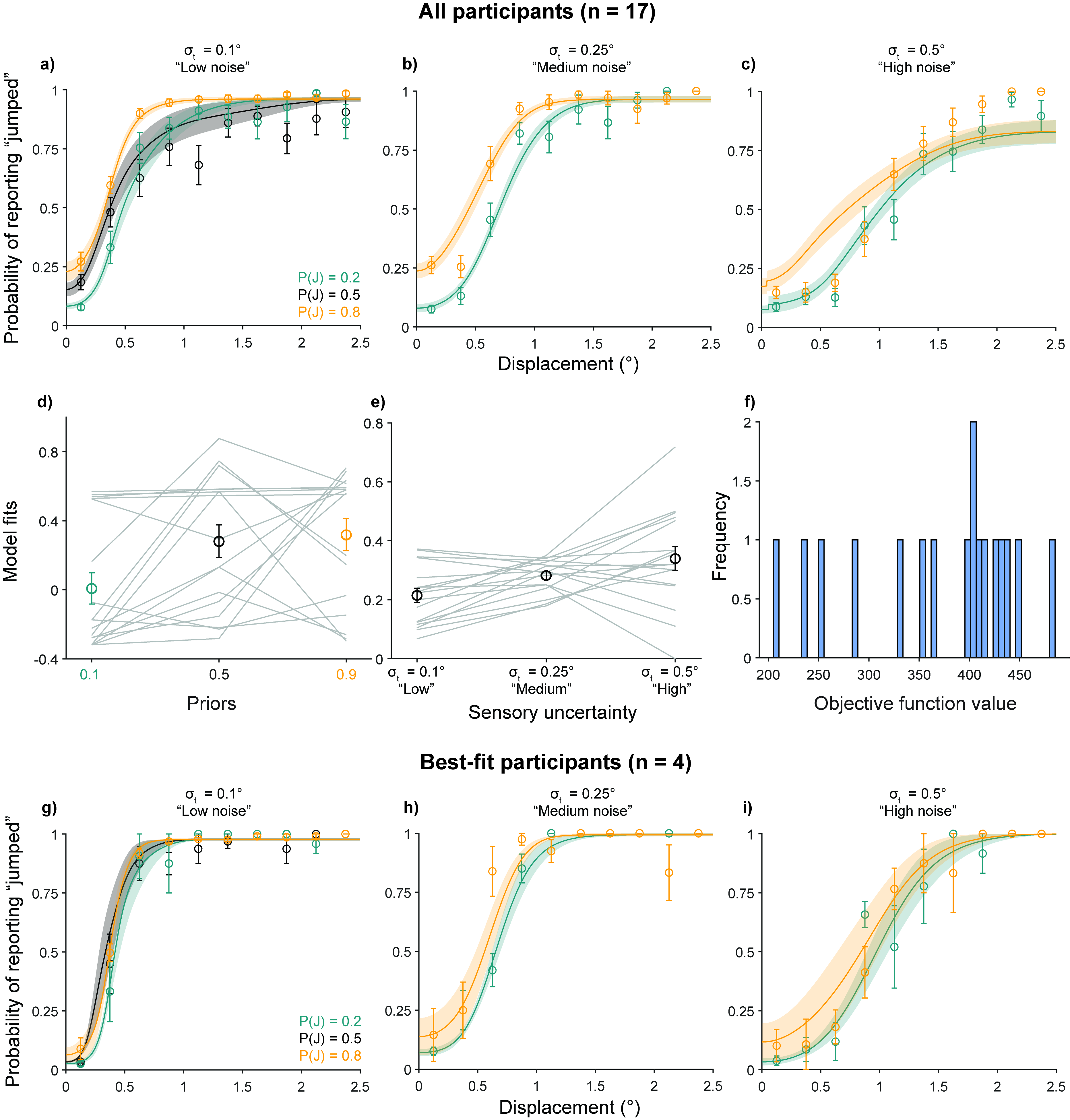

Supplement: Extended Data Figure 4-4 — Bayesian ideal observer model fits in Experiment 2 did not recapitulate the patterns observed in the data. a–c, The model recapitulated the observed patterns in the binned, empirical data reasonably well for the low-noise (a), medium-noise (b) conditions. However, for the high-noise condition (c), it systematically overestimated the probability of reporting “jumped” in the high prior condition, and therefore, prior use. d, e, Fit prior (d) and sensory noise (e) parameter values. f, Histogram of objective function (i.e., model error) values for all participants. g–i, Same as a–c, but for the four participants with the lowest model error values (<300). The same overall pattern of deviation from the data was observed, i.e., the model overestimated prior use in the high-noise condition. Fit lapse rates in the highest noise condition were higher than in the other two conditions (low noise: 0.04 ± 0.009, medium noise: 0.03 ± 0.01, high noise: 0.17 ± 0.05), and the widths of the “jump” distribution (3.03 ± 0.35) were higher than those of the “nonjump” distribution (0.47 ± 0.17) as expected. Download Figure 4-4, TIF file. [file enu-eN-NWR-0403-22-s06.tif]

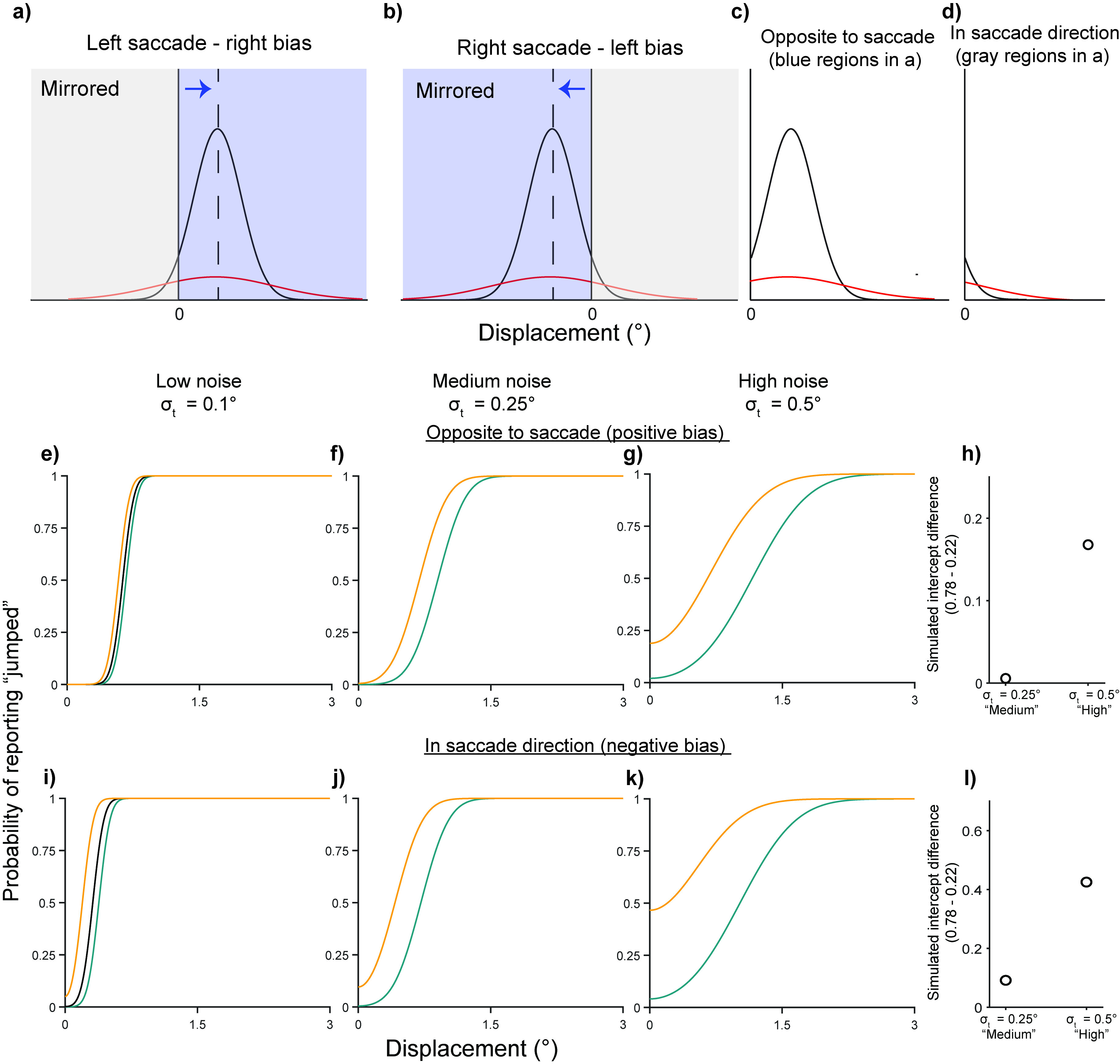

Supplement: Extended Data Figure 5-1 — Incorporating a bias into the categorical Bayesian ideal observer model did not predict the anti-Bayesian results observed in Experiment 2. We modeled an opposite-to-saccade bias for displacements in the categorical Bayesian ideal observer model by shifting the “jump” (red) and “nonjump” (black) distributions from which displacements were drawn. a, For a left saccade, the distributions would shift rightward (indicated by the dashed line) and (b) for a right saccade, they would shift leftward. Since we take the absolute value of displacements to compute our logistic psychometric curves, negative (leftward) displacements and the distributions they are drawn from are mirrored about the y-axis. c, This resulted in a simulated bias >0 for jumps opposite to the saccade (corresponding to the blue shaded regions in a, b) and (d) a bias <0 for jumps in the direction of the saccade (gray regions in a, b). e–g, Simulated psychometric curves with a bias of +0.15° for the (e) low-noise, (f) medium-noise, and (g) high-noise conditions moved further apart with increasing sensory noise. h, Simulated intercept differences in the medium-noise and high-noise conditions (comparable to Fig. 3e) quantified the prediction that prior use increased with increasing noise. i–l, Same as in e–h but with a simulated bias of −15°. Download Figure 5-1, TIF file. [file enu-eN-NWR-0403-22-s07.tif]

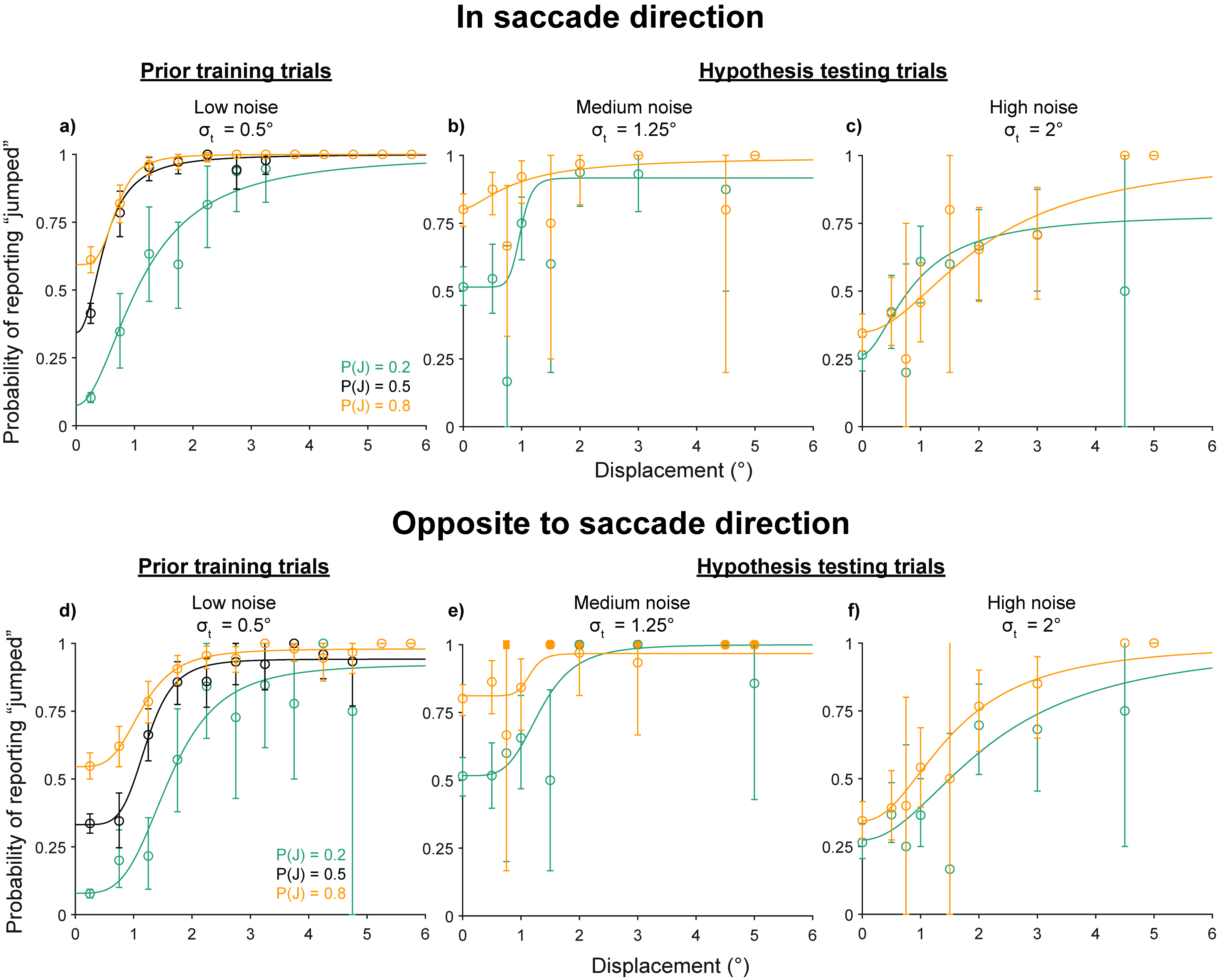

Supplement: Extended Data Figure 7-1 — The direction of target displacement relative to the saccade did not influence the results of Experiment 4 (Monkey S). a–c, Data from displacements in the direction of the saccade. d–f, Data from displacements opposite to the direction of the saccade. a, d, Data from prior learning trials. The direction of target displacement relative to the saccade did not matter for Monkey S, as seen by comparing the data shown here with the pooled data of Figure 7a,d–f. Download Figure 7-1, TIF file. [file enu-eN-NWR-0403-22-s08.tif]

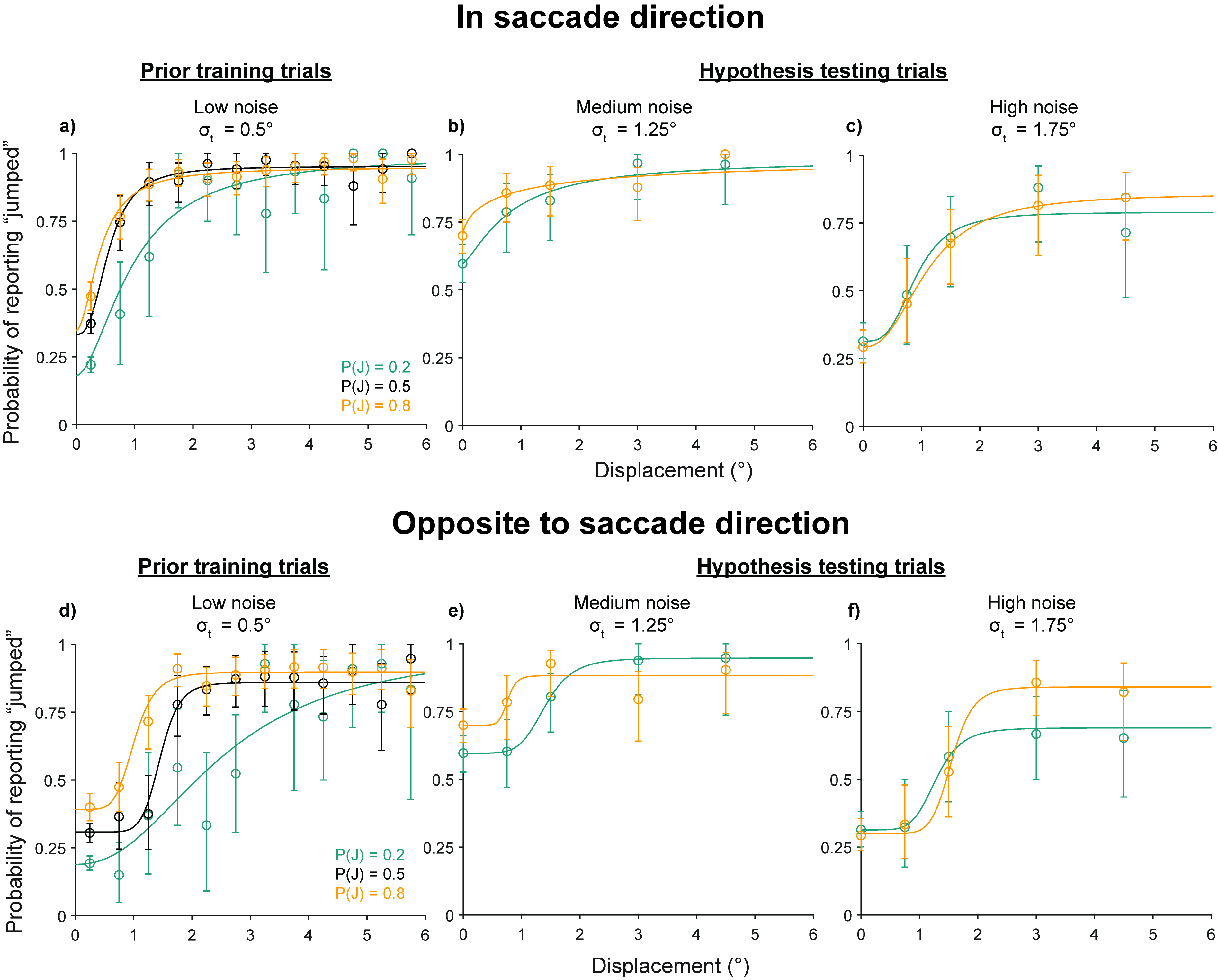

Supplement: Extended Data Figure 7-2 — Same as Extended Data Figure 7-1, but for Monkey T (compare Fig. 7b,g–i). Again, results were essentially unchanged; the direction of target displacement did not matter. Download Figure 7-2, TIF file. [file enu-eN-NWR-0403-22-s09.tif]

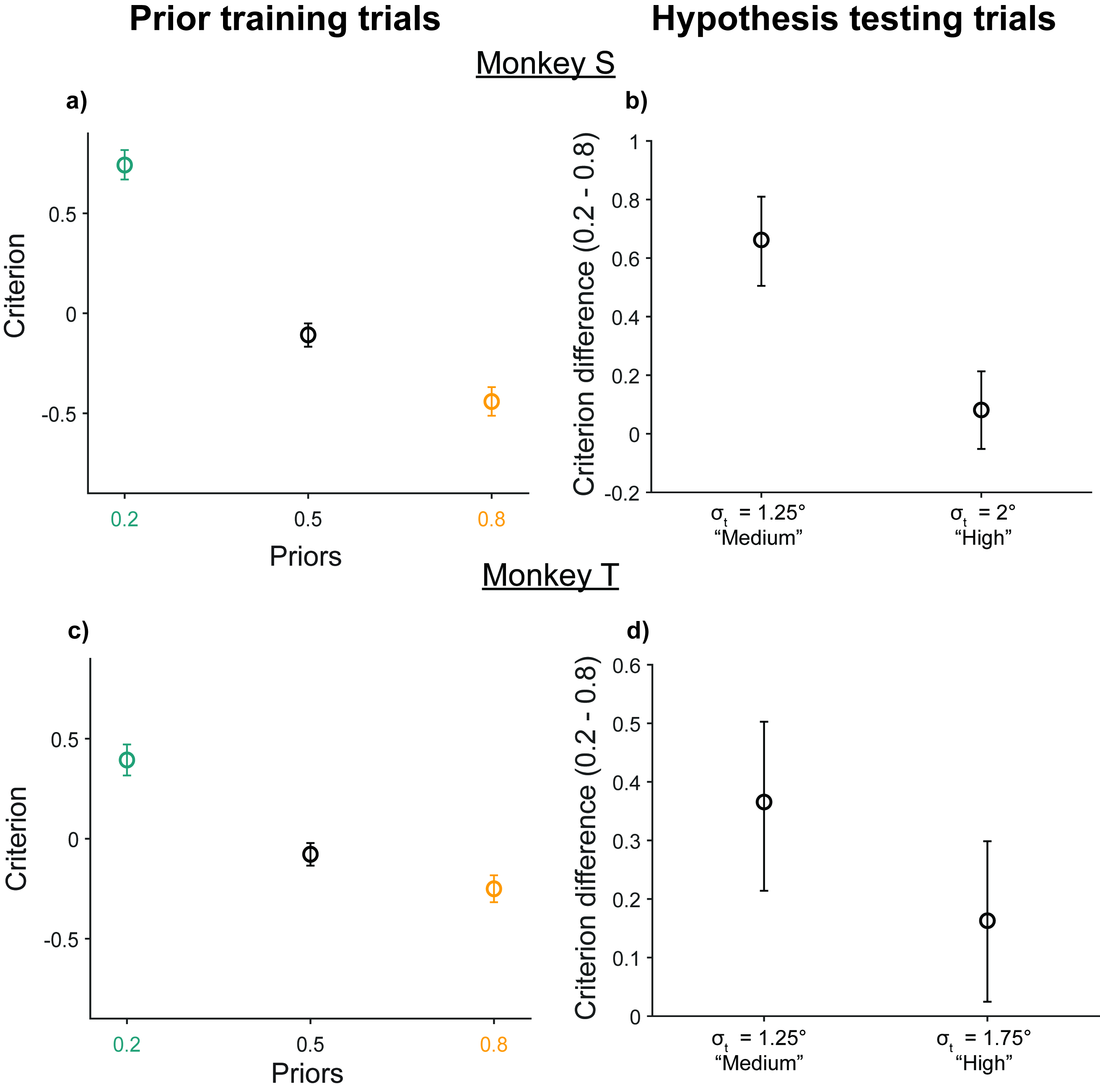

Supplement: Extended Data Figure 7-3 — Replication of the Experiment 4 (image noise) results using Criterion instead of intercepts. a, c, For both monkeys, Criterion decreased with the prior magnitude, demonstrating that they learned the priors in training trials (a: 0.74 [0.67 0.81], −0.11 [−0.17 −0.05], and −0.44 [−0.51 −0.37] for low prior, baseline, and high prior, respectively, for Monkey S, and c: 0.39 [0.32 0.47], −0.08 [−0.13 −0.02], and −0.25 [−0.32 −0.18] for Monkey T). A lower Criterion value meant that participants were more likely to report “jumped.” Plotting conventions as in Figure 7c, except for plotting the results for each monkey separately here. b, d, For both monkeys, Criterion difference between low and high priors in the medium-noise condition (b: 0.67 [0.51 0.81] for Monkey S and d: 0.37 [0.21 0.50] for Monkey T) were higher than in the high-noise condition (b: 0.08 [−0.05 0.21] for Monkey S and d: 0.16 [0.02 0.30] for Monkey T). In other words, the monkeys used their priors less with greater image noise, the same result as when using intercept differences (compare Fig. 7f,i). Plotting conventions as in Figure 7f,i. Download Figure 7-3, TIF file. [file enu-eN-NWR-0403-22-s10.tif]

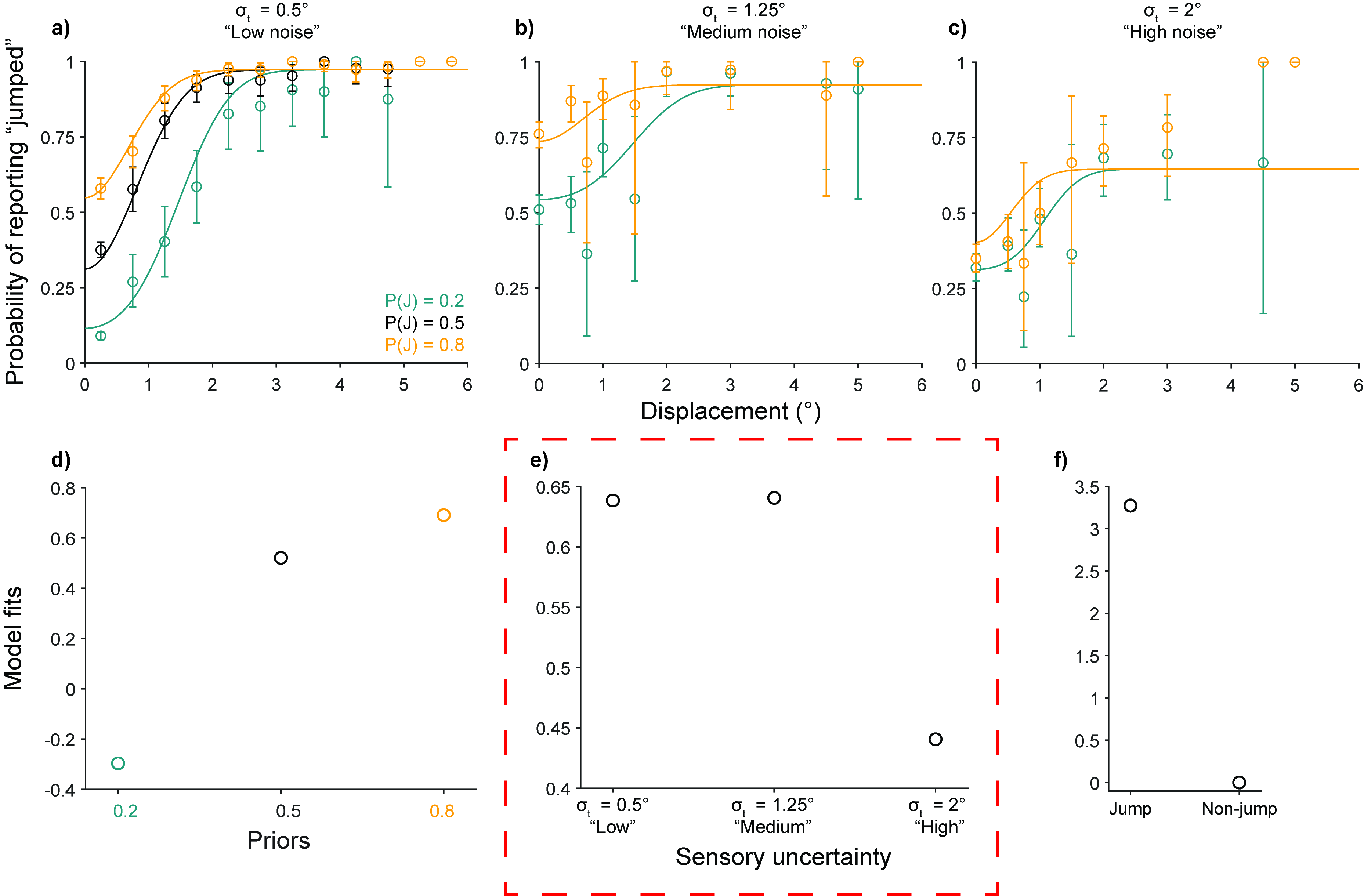

Supplement: Extended Data Figure 7-4 — Bayesian Ideal Observer model fits to data in Experiment 4 produced anti-Bayesian best-fit parameters (Monkey S). a–c, The model recapitulated the observed patterns in the binned, empirical data for the low-noise (a), medium-noise (b), and high-noise (c) conditions. Although the best-fit output parameters increased with increasing priors [d, −0.3, 0.5, and 0.70 for the P(J) = 0.2, P(J) = 0.5, and P(J) = 0.8 conditions, respectively], the output parameters for the sensory noise level were the opposite of those expected by increasing the target blurriness (e, 0.64°, 0.64°, and 0.44° for the low-noise, medium-noise, and high-noise conditions, respectively; highlighted by red dashed box). f, Best-fit parameters for widths of the “jump” (3.27°) and “nonjump” (0.0003°) qualitatively matched the directions of the values used in the experiment. The fit lapse rates increased with sensory noise as expected (for low, medium, and hig, respectively, the fit lapse rates were 0.03, 0.08, and 0.36). Download Figure 7-4, TIF file. [file enu-eN-NWR-0403-22-s11.tif]

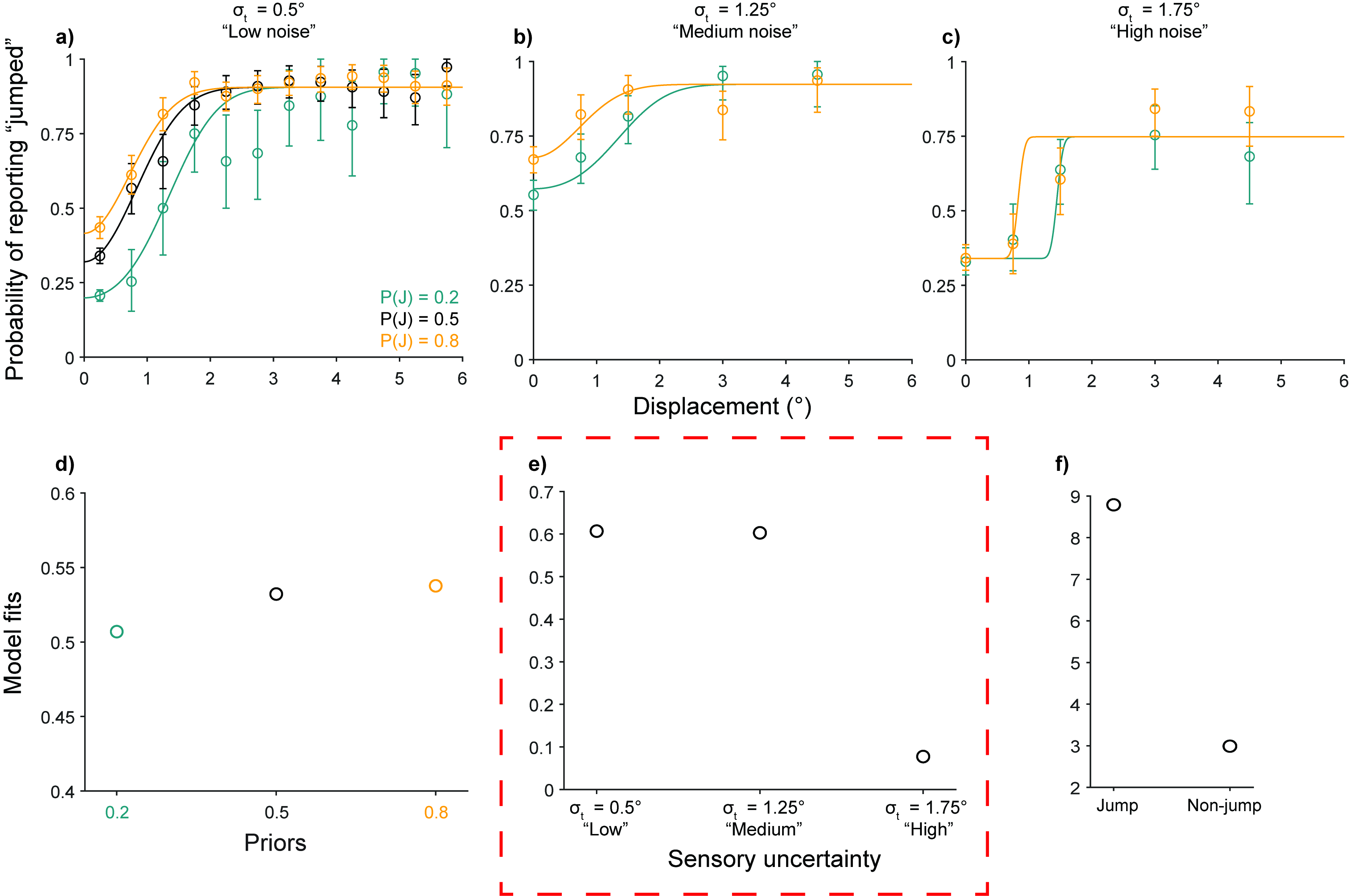

Supplement: Extended Data Figure 7-5 — Same as Extended Data Figure 7-4, but for Monkey T. a–c, Again, the model recapitulated the observed patterns in the binned, empirical data for the low-noise (a), medium-noise (b), and high-noise (c) conditions. Although the best-fit output parameters qualitatively increased with increasing priors [d, 0.51, 0.53, and 0.54 for the P(J) = 0.2, P(J) = 0.5, and P(J) = 0.8 conditions, respectively], the output parameters for the sensory noise level were the opposite of those expected by increasing the target blurriness (e, 0.61°, 0.60°, and 0.08° for the low-noise, medium-noise, and high-noise conditions, respectively; highlighted by red dashed box). f, Best-fit parameters for widths of the “jump” (8.79°) and “nonjump” (2.99°) qualitatively matched the directions of the values used in the experiment. The fit lapse rates largely increased with sensory noise (for low, medium, and high noise, respectively, the fit lapse rates were 0.09, 0.08, and 0.25 for Monkey T). Download Figure 7-5, TIF file. [file enu-eN-NWR-0403-22-s12.tif]

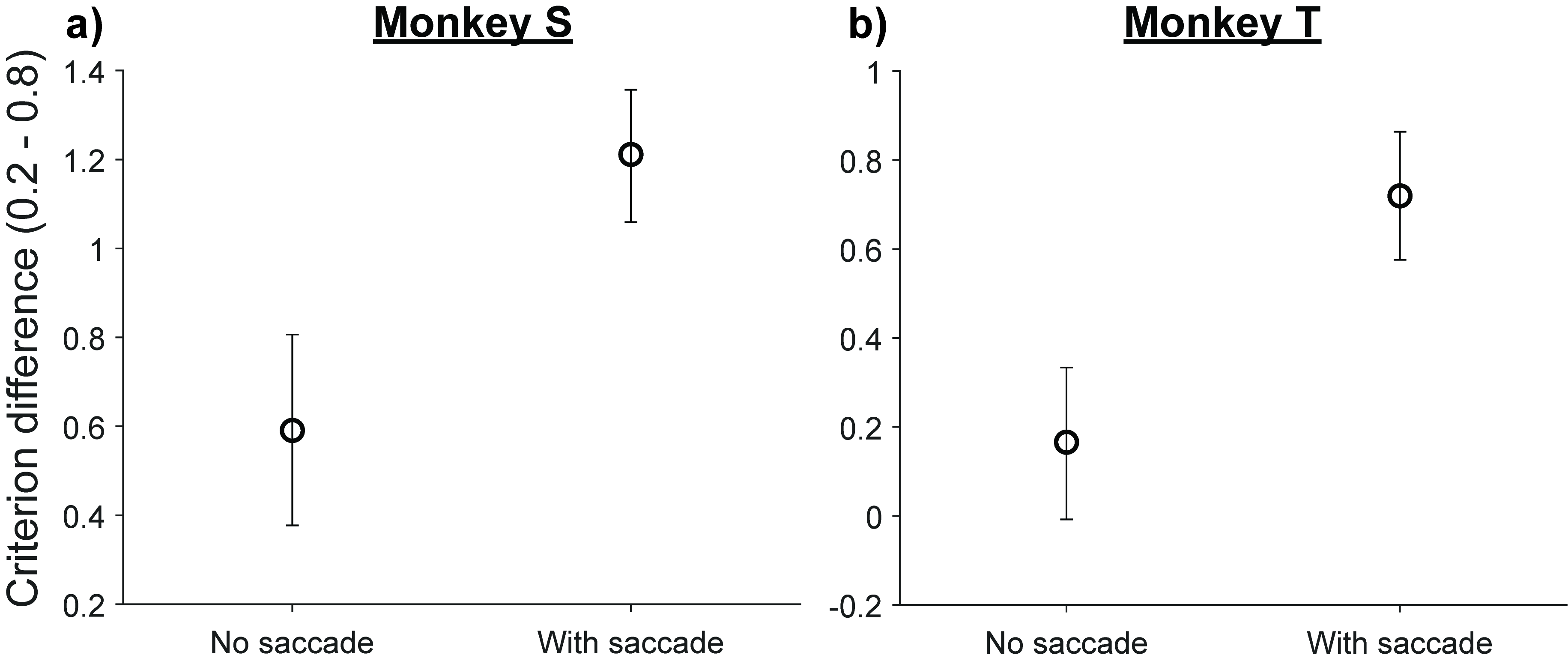

Supplement: Extended Data Figure 8-1 — Replication of the Experiment 5 (motor-driven noise) results using Criterion instead of intercepts. For both Monkey S (a) and Monkey T (b), the Criterion difference in the with-saccade condition (S: 1.21 [1.06 1.36], T: 0.72 [0.58 0.86]) was higher than in the no saccade condition (S: 0.59 [0.38 0.81], T: 0.17 [−0.01 0.33]), indicative of increased prior use with uncertainty and therefore Bayesian behavior. This is the same result found using intercepts (compare Fig. 8f,i). Download Figure 8-1, TIF file. [file enu-eN-NWR-0403-22-s13.tif]

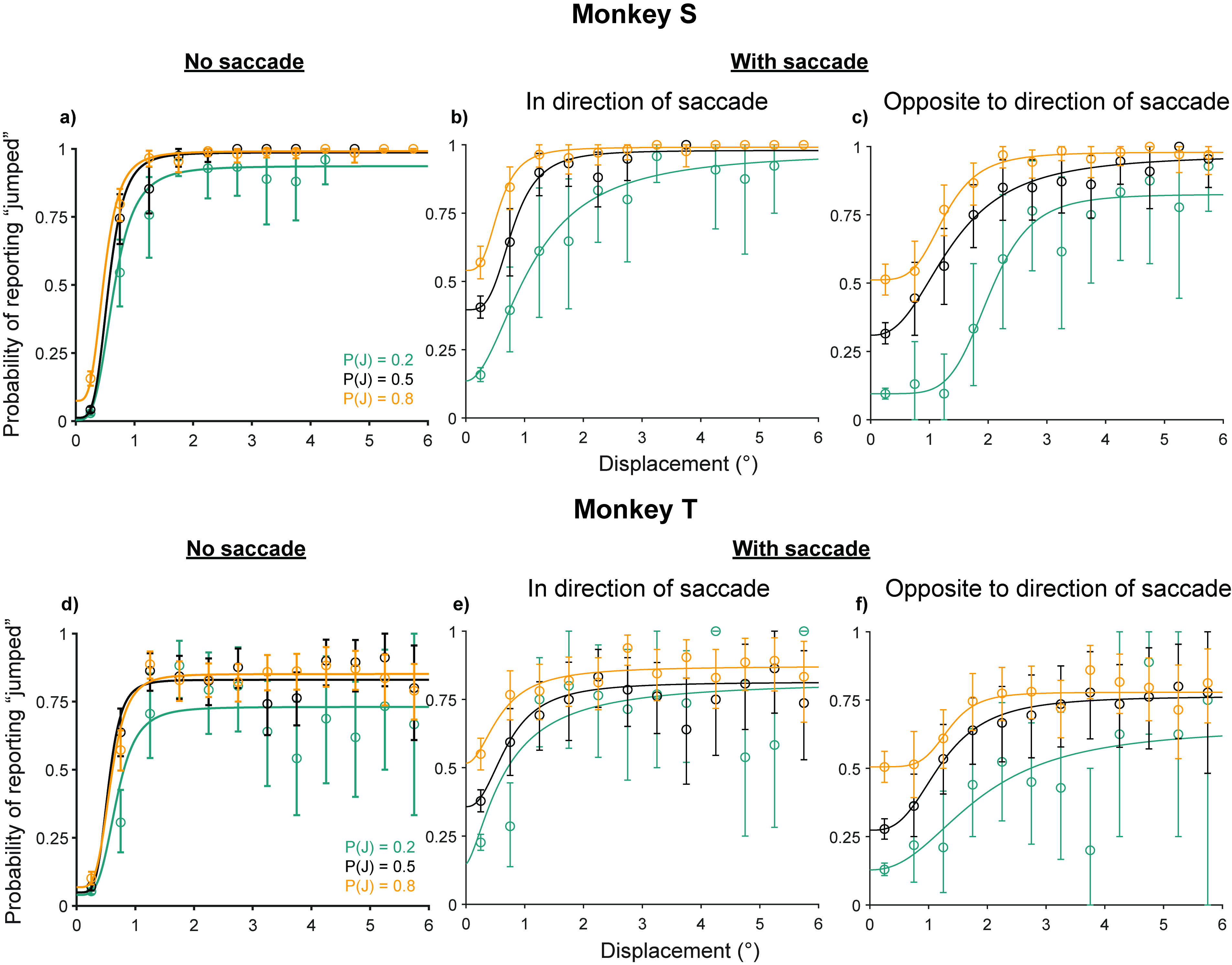

Supplement: Extended Data Figure 8-2 — The direction of target displacement relative to the saccade did not influence the results of Experiment 5 (motor-driven noise). As with the categorical image noise experiments (Extended Data Fig. 3-1), the results did not change when data were split by direction of target displacement relative to the saccade. For both Monkeys S (a–c) and T (d–f), psychometric curves for the different prior conditions were further apart in the with saccade conditions regardless of the direction of the displacement (b, c, e, f) than in the no saccade condition (a, d). That is, they used their priors more when experiencing motor-driven noise than without motor-driven noise. These are essentially the same results as found when the directions of target displacements were pooled (compare Fig. 8d,e,g,h). Download Figure 8-2, TIF file. [file enu-eN-NWR-0403-22-s14.tif]
